# PANX1-mediated ATP release confers FAM3A’s suppression effects on hepatic gluconeogenesis and lipogenesis

**DOI:** 10.1186/s40779-024-00543-6

**Published:** 2024-06-27

**Authors:** Cheng-Qing Hu, Tao Hou, Rui Xiang, Xin Li, Jing Li, Tian-Tian Wang, Wen-Jun Liu, Song Hou, Di Wang, Qing-He Zhao, Xiao-Xing Yu, Ming Xu, Xing-Kai Liu, Yu-Jing Chi, Ji-Chun Yang

**Affiliations:** 1 Department of Physiology and Pathophysiology, School of Basic Medical Sciences/State Key Laboratory of Vascular Homeostasis and Remodeling/Center for Non-Coding RNA Medicine, Peking University Health Science Center, Beijing, 100191 China; 2 Department of Obstetrics and Gynecology, Peking University Third Hospital/National Clinical Research Center for Obstetrics and Gynecology, Beijing, 100191 China; 3 Department of Endocrinology, Beijing Chao-Yang Hospital, Capital Medical University, Beijing, 100020 China; 4 Department of Central Laboratory and Institute of Clinical Molecular Biology, Peking University People’s Hospital, Beijing, 100044 China; 5 Department of Gastroenterology, Peking University People’s Hospital, Beijing, 100044 China; 6 Department of Cardiology, Institute of Vascular Medicine, Peking University Third Hospital/Key Laboratory of Molecular Cardiovascular Science of the Ministry of Education, Beijing, 100191 China; 7 Department of Hepatobiliary and Pancreatic Surgery, General Surgery Centre, the First Hospital of Jilin University, Changchun, 130061 China; 8 Department of Cardiology, Peking University Third Hospital, Beijing, 100191 China

**Keywords:** Pannexin 1 (PANX1), Family with sequence similarity 3 member A (FAM3A), Adenosine triphosphate (ATP) release, Glucolipid metabolism

## Abstract

**Background:**

Extracellular adenosine triphosphate (ATP) is an important signal molecule. In previous studies, intensive research had revealed the crucial roles of family with sequence similarity 3 member A (FAM3A) in controlling hepatic glucolipid metabolism, islet β cell function, adipocyte differentiation, blood pressure, and other biological and pathophysiological processes. Although mitochondrial protein FAM3A plays crucial roles in the regulation of glucolipid metabolism via stimulating ATP release to activate P2 receptor pathways, its mechanism in promoting ATP release in hepatocytes remains unrevealed.

**Methods:**

*db/db*, high-fat diet (HFD)-fed, and global pannexin 1 (*PANX1*) knockout mice, as well as liver sections of individuals, were used in this study. Adenoviruses and adeno-associated viruses were utilized for in vivo gene overexpression or inhibition. To evaluate the metabolic status in mice, oral glucose tolerance test (OGTT), pyruvate tolerance test (PTT), insulin tolerance test (ITT), and magnetic resonance imaging (MRI) were conducted. Protein–protein interactions were determined by coimmunoprecipitation with mass spectrometry (MS) assays.

**Results:**

In livers of individuals and mice with steatosis, the expression of ATP-permeable channel PANX1 was increased (*P* < 0.01). Hepatic PANX1 overexpression ameliorated the dysregulated glucolipid metabolism in obese mice. Mice with hepatic *PANX1* knockdown or global *PANX1* knockout exhibited disturbed glucolipid metabolism. Restoration of hepatic PANX1 rescued the metabolic disorders of PANX1-deficient mice (*P* < 0.05). Mechanistically, ATP release is mediated by the PANX1-activated protein kinase B-forkhead box protein O1 (Akt-FOXO1) pathway to inhibit gluconeogenesis via P2Y receptors in hepatocytes. PANX1-mediated ATP release also activated calmodulin (CaM) (*P* < 0.01), which interacted with c-Jun N-terminal kinase (JNK) to inhibit its activity, thereby deactivating the transcription factor activator protein-1 (AP1) and repressing fatty acid synthase (FAS) expression and lipid synthesis (*P* < 0.05). FAM3A stimulated the expression of PANX1 via heat shock factor 1 (HSF1) in hepatocytes (*P* < 0.05). Notably, FAM3A overexpression failed to promote ATP release, inhibit the expression of gluconeogenic and lipogenic genes, and suppress gluconeogenesis and lipid deposition in PANX1-deficient hepatocytes and livers.

**Conclusions:**

PANX1-mediated release of ATP plays a crucial role in maintaining hepatic glucolipid homeostasis, and it confers FAM3A’s suppressive effects on hepatic gluconeogenesis and lipogenesis.

**Supplementary Information:**

The online version contains supplementary material available at 10.1186/s40779-024-00543-6.

## Background

According to the latest report of the International Diabetes Federation in 2021, diabetes has emerged as a widespread global concern, affecting approximately 10.5% of the global population [1]. It is estimated that there are currently 537 million people with diabetes, with this number expected to rise to 783 million by 2045 [1, 2]. Diabetes is highly associated with non-alcoholic fatty liver disease (NAFLD), obesity, and cardiovascular disorders [3, 4]. The liver is the central organ responsible for regulating glucolipid metabolism in response to insulin and other hormones or physiological factors [5]. Excessive hepatic gluconeogenesis and lipogenesis are the key factors that contribute detrimentally to hyperglycemia, hypertriglyceridemia, diabetes, and NAFLD [6].

Within cells, adenosine triphosphate (ATP) is synthesized by mitochondrial ATP synthase (ATPS) and serves as the most important energy storage molecule [7]. Reduced ATP synthesis in the liver is strongly associated with metabolic disorders in both humans and animals [8–11]. Emerging evidence has also suggested that ATP and its metabolic products function as important extracellular signaling molecules [12]. Released ATP can be decomposed into adenosine diphosphate, adenosine monophosphate, and adenosine. These molecules exert important biological functions by binding to the purinergic receptors (PRs) on the plasma membrane to activate the downstream signaling pathways [13]. PRs can be classified into P1R, primarily activated by adenosine, and P2R, which can be further subdivided into P2X and P2Y subtypes. Seven P2X receptors (P2X1-7), predominantly activated by ATP, function as ion-channel receptors, while 8 P2Y receptors (P2Y1, P2Y2, P2Y4, P2Y6, P2Y11, P2Y12, P2Y13, P2Y14), activated mainly by ATP, adenosine diphosphate, or uridine triphosphate, are G-protein-coupled receptors [12, 14].

The family with sequence similarity 3 (*FAM3*) gene family is a cytokine-like gene family, which comprises 4 members called *FAM3A-D*, respectively [15]. In the past decade, intensive researches conducted by our and others’ groups have revealed the crucial roles played by members of the *FAM3* gene family in regulating hepatic glucolipid metabolism, islet β cell function, adipocyte differentiation, blood pressure, and other biological and pathophysiological processes [16–24]. Particularly, FAM3A promotes ATP synthesis via direct interaction with the F1 component of F1Fo-ATP synthase [16, 17]. Subsequently, the secreted ATP activates the P2 receptor-protein kinase B-DNA-binding transcriptional regulator CreB-forkhead box D3 (Akt-CREB-FOXD3) pathway to regulate the assembly and capacity of ATPS, maintaining metabolic homeostasis [16, 17]. Activation of FAM3A by doxepin or imipramine treatment markedly ameliorated hyperglycemia, liver steatosis, and obesity in obese mice [22, 25]. Notably, the regulatory effects of doxepin or imipramine on glucolipid metabolism were lost in FAM3A-null mice. Overall, the current findings have established FAM3A as a viable and exciting target for treating metabolic diseases. However, although we have revealed that FAM3A’s suppression effects on hepatic gluconeogenesis and lipid deposition are dependent on ATP release, the mechanism(s) underlying how it regulates ATP release in hepatocytes remains unrevealed, which hinders a comprehensive understanding of its regulatory mechanism(s) and the development of new activators for diseases treatment.

ATP release can occur through multiple pathways in various cell types, including exocytosis, plasma membrane-derived microvesicles, maxi-anion channels, volume-regulated anion channels, calcium homeostasis modulator 1, and membrane channels such as connexin (CX) and pannexin (PANX) [26]. While rapid exocytosis of ATP-enriched vesicles has been demonstrated to facilitate ATP release, sustained ATP release primarily relies on membrane ATP transport [27]. Recently, certain CX and PANX membrane transporters have been found to allow the passage of ATP in various cell types [28, 29]. Although CXs and PANXs share similar structural features, PANXs mainly form hemichannels without participating in gap junctions [30]. Nevertheless, one study also observed that pannexin 1 (PANX1) could perform as the cell–cell channel in vertebrate cells [31]. The CX family comprises 21 and 20 members in human and mouse, respectively. In contrast, 3 members of the PANX family, designated as PANX1, PANX2, and PANX3, are functionally expressed in mammals. Among the numerous CX and PANX members, CX26, CX30, CX32, CX43, CX46, CX50, and PANX1 have been identified to be involved in the process of ATP release across diverse cell types or organs [32–34]. Despite the well-elucidated signaling cascades upon activation of the ATP-P2 receptor axis, the mechanisms underlying ATP release from specific cell types are still largely elusive [32]. Furthermore, the distribution of CXs and PANXs, particularly their roles in the regulation of glucolipid metabolism in hepatocytes remains unclear.

**Fig. 1:**
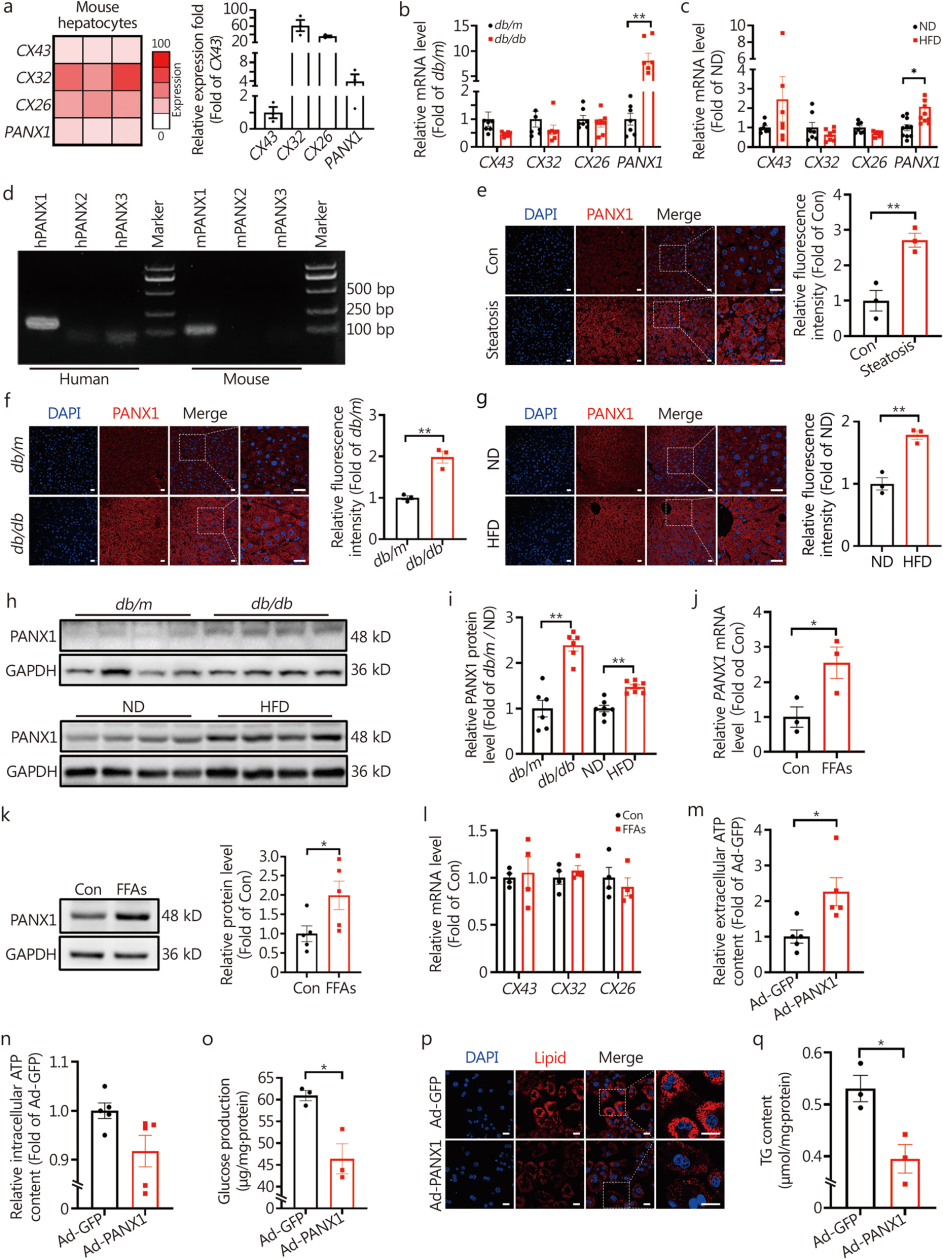
Pannexin 1 (PANX1)-mediated ATP release repressed glucose production and lipid deposition in cultured hepatocytes. **a** Determining the expression profile of *CXs* and *PANXs* using RNA-sequencing in primary hepatocytes. The left panel showed the heat map based on transcripts per million (TPM), and the relative expression fold of *CXs* and *PANXs* was displayed in the right panel (*n* = 3). Change in *CXs* and *PANX1* mRNAs in *db/db* (**b,** *n* = 7) and high-fat diet (HFD)-fed mouse livers (**c**, from left to right, *n* = 7, 7, 7, 7, 7, 7, 10 and 10, respectively). **d** Representative agar gel image of *PANX* expressions in HepG2 cells and mouse hepatocytes. Representative immunofluorescent staining images of PANX1 expression in the livers of patients with non-alcoholic fatty liver disease (NAFLD) (**e**), *db/db* (**f**), and HFD-fed mice (**g**) were shown in the left panel, and quantitative data displayed in the right panel (*n* = 3). Scale bar = 25 μm. PANX1 protein was increased in obese mouse livers as determined by Western blotting assay. Representative gel images of PANX1 protein level in the livers of *db/db* (upper) and HFD-fed (lower) mice were displayed in panel (**h**), and quantitative data were displayed in panel (**i**), from left to right, *n* = 6, 6, 7 and 7, respectively. **j** FFAs treatment elevated the *PANX1* mRNA level in mouse hepatocytes (*n* = 3). **k** FFAs treatment elevated PANX1 protein level in mouse hepatocytes. The left panel represented representative gel images, while the right panel showed the quantitative data (*n* = 5). **l** Exposure to FFAs failed to influence the *CXs* mRNA levels in mouse hepatocytes (*n* = 4). The effects of PANX1 overexpression on extracellular (**m**) and intracellular (**n**) ATP content in mouse hepatocytes (*n* = 5). **o** PANX1 overexpression reduced glucose production in mouse hepatocytes (*n* = 3). **p** and** q** PANX1 overexpression decreased FFAs-induced lipid deposition in mouse hepatocytes. The panel (**p**) displayed representative images of lipid staining, while the panel (**q**) showed quantitative TG content (*n* = 3). Scale bar = 25 μm. ^*^P < 0.05 or ^**^P < 0.01. CXs connexins, PANXs pannexins, ND normal diet, Con control, FFAs free fatty acids, Ad-GFP Ad-GFP-infected hepatocytes, Ad-PANX1 Ad-PANX1-infected hepatocytes, TG triglyceride, DAPI 4,6-diamino-2-phenyl indole

Based on our preliminary RNA sequencing experiment characterizing the expression profile of CX and PANX in hepatocytes (Fig. 1a), the present study sought to elucidate the role and mechanism of PANX1 in the modulation of hepatic glucolipid metabolism. Furthermore, whether PANX1 was involved in FAM3A-mediated ATP release, and regulation of glucolipid metabolism had also been investigated.

## Methods

### Human liver samples

Liver samples were procured from surgical resections of patients with non-neoplastic liver diseases in 2020, distinguishing between those with NAFLD and non-NAFLD. The collection was carried out in accordance with ethical approval from the Research Ethics Committee of Peking University People’s Hospital (2020PHB337-01). This research project on NAFLD aimed to include adults diagnosed with NAFLD based on sonographic evidence of hepatic steatosis, while excluding individuals with significant alcohol consumption history, other liver diseases, and certain medications known to induce hepatic steatosis. Exclusion criteria also encompass pregnant or lactating women, individuals with significant comorbidities, and those not meeting the specified inclusion criteria. All NAFLD patients displayed histologic evidence of hepatic steatosis. Further details regarding the clinical parameters of individuals were presented in Additional file 1: Table S1.

### Experimental animals

Male (8 to 10 weeks old) C57BL/6J mice were fed on either a normal diet (ND) or 45% high-fat diet (HFD) obtained from Medicience (#MD12032, China) for 3 months, respectively. Male *db/db* mice aged 10 to 12 weeks, with a C57BLKS/J (BKS) background, were utilized in this study. All animals acclimated to standard laboratory conditions (12 light: 12 dark cycle) at (24 ± 2) °C. *PANX1* knockout mice were generated by CRISPR/Cas9 technology and purchased from Jiangsu Gem Pharmatech Co., Ltd, China. Approval for all animal experiments was granted by the Institutional Animal Care and Use Committee of Peking University Health Science Center (LA2018179).

### RNA sequencing

Total RNA was extracted from mouse primary hepatocytes using TRIzol® Reagent according to the manufacturer’s instructions. Then RNA quality was determined by 5300 Bioanalyser (Agilent) and quantified using the ND-2000 (NanoDrop Technologies). Only high-quality RNA samples [OD_260_/_280_ = 1.8 – 2.2, OD_260_/_230_ ≥ 2.0, RNA integrity number (RIN) ≥ 6.5, 28S:18S ≥ 1.0, > 1 μg] were used to construct the sequencing library. RNA purification, reverse transcription (1 μg), library construction, and sequencing were performed at Shanghai Majorbio Bio-pharm Biotechnology Co., Ltd. (Shanghai, China) according to the manufacturer’s instructions (Illumina, San Diego, CA). The initial paired-end reads underwent trimming and quality assessment using fastp with its default settings. Subsequently, the resulting clean reads were individually aligned to the reference genome employing HISAT2 software in orientation mode. The mapped reads from each sample were then subjected to assembly using StringTie. Transcript expression levels were determined utilizing the transcripts per million reads method. Gene abundances were quantified using RNA-Seq by Expectation–Maximization (RSEM) (Additional file 2: Table S1).

### Cell culture and treatments

Mouse primary hepatocyte isolation was performed as previously described [16] and cultured in RPMI-1640 supplied with 10% FBS. HepG2 cells and HEK 293 T cells were cultured in a DMEM (high glucose) medium supplied with 10% FBS. All cells were cultured at 37 °C in a humidified atmosphere consisting of 5% CO_2_ and 95% air. Firstly, the multiplicity of infection for adenovirus (Ad) infection was determined based on dose–response analysis, and then mouse primary hepatocytes and cell lines were infected with 50 multiplicities of infection of Ad-GFP, Ad-PANX1, Ad-FAM3A or Ad-CaM for 24 h. For inhibition of PANX1, P2 receptor and HSF1, the cells were treated with probenecid (PBN, PANX1 inhibitor, 200 μmol/L, #S4022, Selleck, China) [35], suramin (50 μmol/L, #ab120422, Abcam, USA) [22], PPADS (50 μmol/L, #HY-101044, MedChemExpress, USA) [36], MRS2179 (10 μmol/L, #HY-101308A, MedChemExpress, USA) [20], PSB-0739 (10 μmol/L, #HY-108660, MedChemExpress, USA) [37] or KRIBB11 (1 μmol/L, #S8402, Selleck, China) [38] for 24 h, respectively. To observe the effect of PBN on ATP release mediated by FAM3A and PANX1, the cells were treated with Ad-FAM3A or Ad-PANX1 for 24 h and then washed twice with 37 °C prewarmed PBS, and subsequently cultured with fresh RPMI-1640 supplemented with 10% FBS and PBN (200 μmol/L) for 2 h.

### Antibodies

Anti-PANX1 antibody (#12,595–1-AP) was purchased from Proteintech (China). Anti-p-Akt (Ser473, #4060S), Akt (#9272S), forkhead box protein O1 (FOXO1, #2880S), p-FOXO1 (Ser256, #9461S), phosphorylated c-Jun N-terminal kinase (p-JNK, Thr183/Thr185, #4668S) and heat shock factor 1 (HSF1, #4356S) antibodies were obtained from Cell Signaling Technology (USA). Anti-glucose-6-phosphatase (Anti-G-6-Pase, #sc-25840) and phosphoenolpyruvate carboxykinase, (PEPCK, #BS6870) antibodies were obtained from Santa-Cruz Biotechnology (USA) and Bioworld (China), respectively. Anti-JNK (#YT2440), activator protein-1 (AP1, #YM0027), and v-maf musculoaponeurotic fibrosarcoma oncogene homolog K (MafK, #YN2091) antibodies were obtained from Immunoway (China). Anti-p-AP1 (Ser63, #ab32385) and fatty acid synthase (FAS, #ab22759) antibodies were purchased from Abcam (USA). Anti-FAM3A (#A2784) and calmodulin (CaM, #PTM-6314) antibodies were obtained from ABclonal (China) and PTM-BIO (China), respectively. Anti-GAPDH antibody (#TA-08) was obtained from ZSGB-BIO (China) and served as a control. All primary antibodies were applied at a 1:1000 dilution for Western blotting. Secondary antibodies, purchased from Biodragon (China), were incubated at a 1:5000 dilution for Western blotting. Meanwhile, all secondary antibodies for immunofluorescence, obtained from ZSGB-BIO (China), were utilized with a dilution of 1:200.

### Adenovirus- and adeno-associated virus (AAV)-mediated overexpression of PANX1 and FAM3A in mouse livers

Male C57BL/6J mice were subjected to either an ND or HFD for 3 months. For this investigation, male *db/db*, HFD mice, or *PANX1* knockout mice received tail vein injections of 1.0 × 10^9^ pfu Ad-green fluorescence protein (GFP) or Ad-PANX1, respectively. Subsequently, these mice underwent different tests to monitor metabolic phenotypes and were sacrificed within 2 weeks post-adenovirus injection. The AAV serotype 8 with the cytomegalovirus (CMV) promoter (AAV-GFP and AAV-FAM3A) was constructed by WZ Biosciences Inc., (China) and 5 × 10^11^ vg AAV-GFP or AAV-FAM3A administered into the tail veins of male *PANX1* knockout mice. Magnetic resonance imaging (MRI) was used to quantify body fat and liver fat content, following the protocol described previously [22].

### Liver-specific *PANX1* gene knockdown

The AAV serotype 8 with the human thyroxine-binding globulin (TBG) promoter (AAV-Scramble and AAV-shPANX1) was constructed by Beijing Likely Biotechnology. Subsequently, male C57BL/6J mice received tail vein injections of 5 × 10^11^ vg AAV-Scramble or AAV-shPANX1, respectively. These mice were then fed on an ND or HFD and performed different tests to monitor metabolic phenotypes within 3 months after injection. MRI was used to quantify body fat and liver fat content, following the protocol described previously [22].

### Oral glucose tolerance test (OGTT), insulin tolerance test (ITT), and pyruvate tolerance test (PTT) assays

OGTT, ITT, and PTT were performed at indicated times in different animal models. Detailed protocols for these tests were introduced previously [39].

### Total triglyceride (TG) assays

TG Determination Kit (#E1013, Applygen, China) was used to measure the TG content in tissues and cells [40]. The content of TG was normalized by the content of protein in the same sample. Serum TG level was measured with another Kit (#E1002) from the same company.

### Plasmid and siRNA transfection

Different plasmids (pcDNA3.1, pGFP, pHSF1, pCaM, pMafK, pGL-3, and pRL-TK) were transfected for 24 h using Quickshuttle transfection reagent (#KX0110042, Biodragon, China) to the mouse primary hepatocytes and cell lines according to the instructions. For *MafK* knockdown, specific siRNAs targeting *MafK* were synthesized by Shanghai Yaoyuan Jingdian Biotechnology Co., Ltd., China. The sequences were provided in Additional file 1: Table S2. Cells were administrated with 100 nmol/L si-MafK or control sequence for 24 h using the same transfection reagent.

### Luciferase reporter assay

Mouse *PANX1* gene promoter sequence (-2000 bp to 0 bp) and mouse *FAS* gene sequence (-2000 bp to 250 bp) were cloned into the pGL3-basic vector, respectively. *PANX1* gene promoter was co-transfected with HSF1 or MafK or GFP expressing plasmid or pcDNA3.1 in HepG2 cells or HEK 293 T cells using Quickshuttle transfection reagent according to the instructions. *FAS* gene promoter was co-transfected with AP1 or CaM or GFP expressing plasmid or pcDNA3.1 in HepG2 cells using the same transfection reagent. Meanwhile, the pRL-TK vector was co-transfected in each condition as a control. Luciferase activities were measured after 24 h using the Dual-Luciferase Reporter Kit (#T002, Vigorous Biotechnology, China).

### ATP content determination

ATP-Lite Assay Kit (#T007, Vigorous Biotechnology, China) and the Luminometer (Turner Biosystems, USA) were used to determine ATP with a bioluminescence assay as detailed previously [23]. Standard ATP samples were used to make a standard curve. After treatment, cell culture media and cellular components were all collected on ice, respectively. For the determination of intracellular and extracellular ATP content, the ATP content of the samples was determined using the ATP standard curve normalized to protein contents and then normalized to control values. Moreover, when the mice were killed, 100 μl blood from the eyeballs was gently taken, and hemolysis was avoided as far as possible. After centrifugation at 3000 r/min (2 min, 4 °C), the supernatant was used for ATP detection immediately.

### Glucose production assay

Mouse primary hepatocytes or HepG2 cells were infected with Ad-GFP, Ad-PANX1, or Ad-FAM3A for 24 h in the absence or presence of PBN (200 μmol/L) or suramin (50 μmol/L). Glucose production was assayed as detailed previously [41].

### RNA extraction and real-time PCR

Total RNA from cells and tissues was extracted using RNApure High-purity Total RNA Rapid Extraction Kit (#RP1202, BioTeke Corporation, China) and then 1.0 μg RNA was reversely transcribed into cDNA using EasyScript All-in-One First-Strand cDNA Synthesis Kit (#AE341-03, TransGen Biotech, China). cDNA was amplified via real-time PCR using TransStart Tip Green qPCR SuperMix (#AQ141-04, TransGen Biotech, China) on the Agilent Mx3000P Real-Time PCR System (Agilent Technologies, USA). The primer details were provided in Additional file 1: Table S3.

### Co-Immunoprecipitation (Co-IP)

Immunoprecipitation Kit (#26,147, Thermo Fisher Scientific, USA) was used for the Co-IP experiment [42]. Mouse primary hepatocytes were infected with Ad-CaM or Ad-PANX1 to overexpress CaM or PANX1 (Ad-GFP served as the control treatment). Then Co-IP experiments were performed with anti-CaM or -JNK antibodies, and IgG was used as the negative control. The obtained Co-IP proteins were subjected to SDS-PAGE, after silver nitrate staining and mass spectrometry (MS) analysis or Western blotting.

### Western blotting assays

Roth lysis buffer supplemented with fresh protease and phosphatase inhibitors was used to extract proteins from cells or tissues. The concentration of the concentrated gel used in the experiment was 5%, and the concentration of the separation gel was generally 10%. For small molecules with a molecular weight of less than 25 kD, the separation gel concentration was 12.5%. SDS-PAGE was employed to separate 30 – 40 μg of total protein. The proteins underwent separation through SDS-PAGE and were subsequently transferred to a nitrocellulose membrane. Subsequently, the membrane was sealed for 1 h using 5% skim milk prepared with TBST, followed by immunoblotting with a primary antibody (1:1000) targeting the gene of interest. On the following day, the membranes were washed with TBST 3 times, each lasting 8 min. Protein bands were exposed to a corresponding secondary antibody (1:5000), and detection was carried out using a chemiluminescence Kit (#BF06053-500, Biodragon, China). Finally, ImageJ software (version 1.8.0.345) was employed to quantify the protein exposure results, and the protein content was calibrated to the corresponding housekeeping gene.

### Immunofluorescent staining

Liver sections underwent a 30 min baking process at 68 °C, followed by immersion in xylene for 5 min, repeated 3 times. Subsequently, they were sequentially placed in 100%, 95%, 90%, 80%, and 70% ethanol (v/v) for 3 min, respectively. Antigen repair was performed with 200 ml of antigen repair solution at 95 °C for 8 min, followed by cooling to room temperature (RT). Blocking with 1% BSA occurred at RT for 30 min, and the primary antibody (1:100) was added and incubated at 4 °C overnight. On the second day, the corresponding fluorescent secondary antibody (1:200) was added and incubated at RT for 1 h away from light. DAPI (1:5000) nucleation took place for 20 min.

For cell treatment with Ad-PANX1 or Ad-FAM3A, with or without various inhibitors, a 24 h incubation period was employed (Ad-GFP or Ad-null served as controls). The cells were washed with PBS and fixed with 4% paraformaldehyde at RT for 15 min. Permeation of the film with a liquid containing 0.05% Triton X-100 and 0.5% BSA occurred at RT for 10 min. After washing with PBS 2 times, seal with 1% BSA for 30 min. Subsequently, after washing with PBS, cells were incubated with the primary antibody (1:100) at 4 °C overnight. On the second day, the second fluorescent antibodies were added at a ratio of 1:200 and incubated at RT for 1 h. The nucleus was stained with DAPI. All fluorescence images were obtained and quantified using a Leica confocal laser scanning microscope.

### Cells neutral lipid staining

Cells infected with Ad-PANX1 or Ad-FAM3A in the presence of free fatty acids (FFAs, 0.2 mmol/L oleic acid and 0.4 mmol/L palmitic acid) and PBN (200 μmol/L) or suramin (50 μmol/L) for 24 h (Ad-GFP as control treatment). Cells were fixed with 4% paraformaldehyde for 15 min, then stained with LipidTOX™ neutral lipid stain (1:1000; #H34476, Invitrogen, USA). After nuclear staining with DAPI, the cells on coverslips were visualized and quantified with Leica Confocal Laser Scanning Microscopy.

### DNA pull-down assay

Specific primer was synthesized according to -1415 bp to -21 bp upstream sequence of the mouse *PANX1* gene promoter region, and the forward primer was labeled with biotin (Forward primer sequence: 5’-Biotin-CCAGAGTGAGCTGGGTGTTAGAG-3’; Reverse primer sequence: 5’-AGCAGTTTCCGGAAGCGCGCTGAC-3’). Biotin-labeled target DNA fragments were amplified by PCR, and the Kit (#DP209) from TIANGEN (China) was used to purify PCR products. Mouse primary hepatocytes were administrated with FFAs for 24 h, then the Kit (#P1200, China) from Applygen was used to extract nucleoprotein. Nucleoprotein (200 μg), DNA (1 μg), 2 × B&W buffer, and nuclease-free water were mixed and incubated at RT in a rotating mixer for 30 min. Then, streptavidin magnetic beads pre-cleaned with 2 × B&W buffer were added, and mixed at RT in a rotating mixer for 30 min. After incubation, the magnetic beads were washed with 1 × B&W buffer pre-cooled at 4 °C for 3 times. A total of 30 μl of 1 × loading buffer was added to resuspend the magnetic beads, and the mixture was heated at 100 °C for 10 min. The obtained pull-down proteins were subjected to SDS-PAGE, followed by silver nitrate staining and MS analyses.

### Statistical analysis

All data were analyzed using GraphPad Prism 8.0 and expressed as mean ± SEM. For data meeting normal distribution, a *t*-test (two groups) or ANOVA test (multiple groups) was used for data analysis. Data that did not meet the normal distribution were statistically analyzed using the Mann–Whitney *U* test (two groups) and the Kruskal–Wallis *H* rank-sum test (multiple groups).* P*-values < 0.05 were considered statistically significant.

## Results

### PANX1-mediated ATP release reduced glucose production and lipid deposition in cultured hepatocytes

Firstly, RNA-sequencing was performed to determine the expression profile of *CXs* and *PANXs* in mouse hepatocytes. As a result, the significant transcriptional signals of *CX43*, *CX32*, *CX26*, and *PANX1* were detected in mouse hepatocytes (Fig. 1a). To evaluate whether these genes were involved in the pathogenesis of metabolic disorders, their mRNA levels were examined in obese mouse livers. The mRNA level of *PANX1* was increased while those of *CX43*, *CX32,* and *CX26* remained unchanged in both *db/db* and HFD-fed mouse livers (Fig. 1b, c). Moreover, PCR analyses confirmed that *PANX2* and *PANX3* were not significantly expressed in human and mouse hepatocytes (Fig. 1d). Furthermore, elevated levels of PANX1 protein were observed in the livers of patients with NAFLD (Fig. 1e, *P* < 0.01) and diabetic mice (Fig. 1f-i). In cultured mouse (Fig. 1j, k) and human (Additional file 1: Fig. S1a, b) hepatocytes, FFAs treatment increased the *PANX1* mRNA and protein levels. In contrast, treatment with FFAs had little effect on the mRNA levels of *CX43*, *CX32,* and *CX26* (Fig. 1l). To elucidate the role of PANX1 in hepatic glucose and lipid metabolism, PANX1 expressing adenovirus (Ad-PANX1) was constructed and verified in HepG2 cells (Additional file 1: Fig. S1c). Overexpression of PANX1 increased extracellular (*P* < 0.05) but not intracellular ATP content in both mouse and human hepatocytes (Fig. 1m, n, Additional file 1: Fig. S1d, e). PANX1 overexpression reduced gluconeogenesis and lipid deposition in both mouse hepatocytes (Fig. 1o-q) and HepG2 cells (Additional file 1: Fig. S1f-h). Collectively, PANX1-mediated ATP release regulates glucolipid metabolism in cultured hepatocytes.

### Hepatic PANX1 played crucial roles in maintaining glucolipid metabolism

**Fig. 2:**
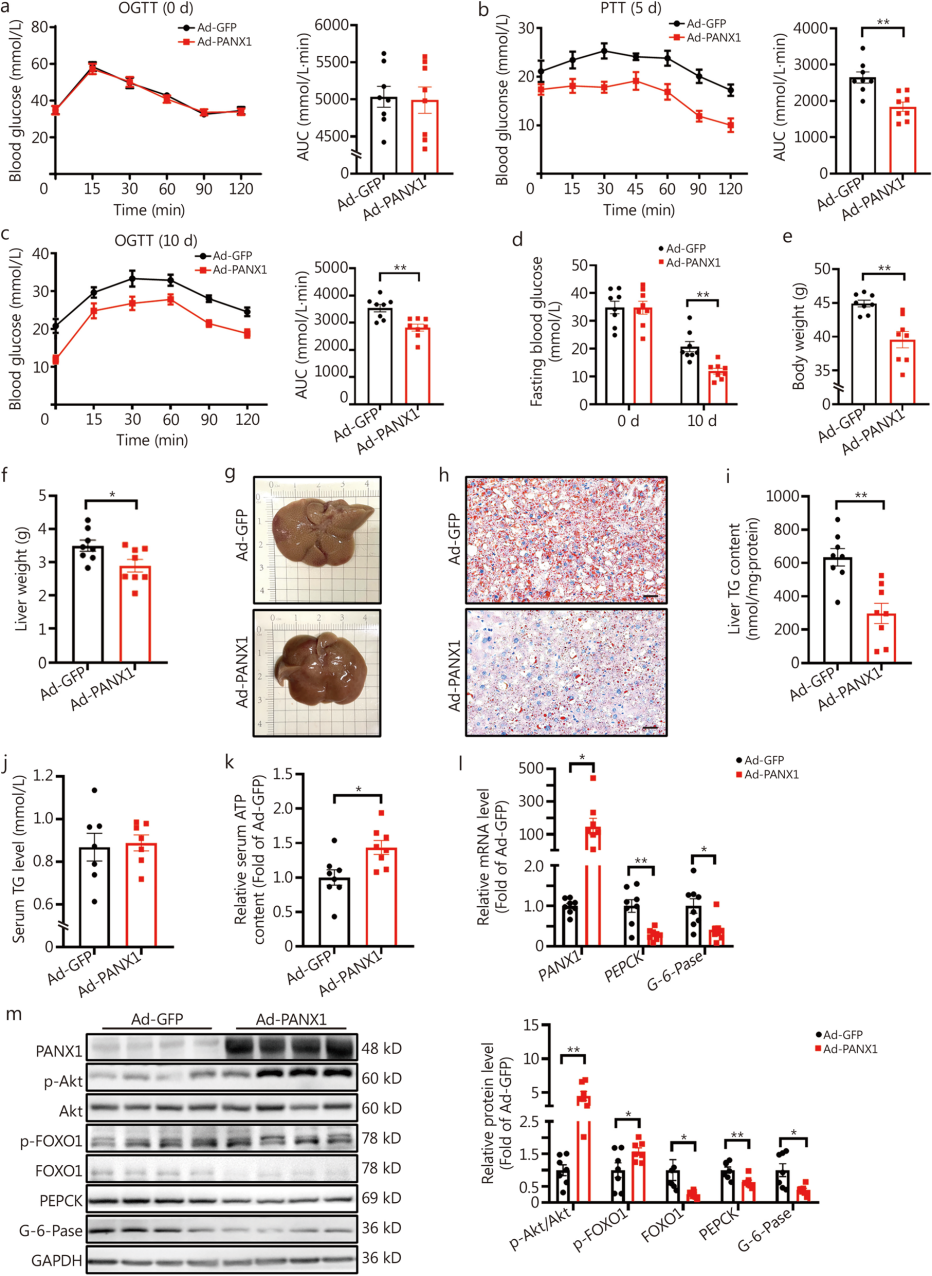
Hepatic pannexin 1 (PANX1) overexpression attenuated hyperglycemia and alleviated fatty liver in *db/db* mice. **a** Oral glucose tolerance test (OGTT) exhibited no significant difference between the two sets of mice before adenoviral injection. The left panel displayed the OGTT curves, while the right panel presented the data for the area under the curve (AUC) (*n* = 8). **b** Pyruvate tolerance test (PTT) curves on day 5 post adenoviral injection were presented in the left panel, and AUC data were displayed in the right panel (*n* = 8). **c** The left panel displayed the OGTT curves on day 10 post adenoviral injection, while the right panel presented the data for the AUC (*n* = 8). **d** Hepatic PANX1 overexpression reduced fasting blood glucose level of *db/db* mice on day 10 after adenoviral injection (*n* = 8). Body weight (**e**) and liver weight (**f**) on day 11 post adenoviral injection (*n* = 8). Morphological observation (**g**) and oil red O staining analysis (**h**) of livers from Ad-GFP and Ad-PANX1-injected *db/db* mice (*n* = 5). Scale bar = 50 μm. Liver (**i**, *n* = 8) and serum (**j**, *n* = 7) triglyceride (TG) content of Ad-GFP and Ad-PANX1-injected *db/db* mice. **k** Serum adenosine triphosphate (ATP) content was increased after PANX1 overexpression in the liver of *db/db* mice. Serum ATP content was detected immediately after sacrifice (*n* = 8). **l** Comparative mRNA levels of gluconeogenic genes in *db/db* mice livers (*n* = 8). **m** The left panel displayed representative gel images of gluconeogenic proteins in *db/db* mouse livers, while the right panel presented quantitative data (*n* = 7). ^*^P < 0.05 or ^**^P < 0.01. Ad-GFP Ad-GFP-injected *db/db* mice, Ad-PANX1 Ad-PANX1-injected *db/db* mice, Akt protein kinase B, FOXO1 forkhead box protein O1, PEPCK phosphoenolpyruvate carboxykinase, G-6-Pase glucose-6-phosphatase

To evaluate the roles of hepatic PANX1 in glucolipid metabolism in vivo, two sets of *db/db* mice were treated with either Ad-GFP or Ad-PANX1, respectively (Fig. 2a). The injection of Ad-PANX1 markedly reduced hepatic glucose production and improved glucose intolerance in *db/db* mice (Fig. 2b, c, *P* < 0.01). Injection of Ad-PANX1 also ameliorated fasting hyperglycemia in mice on day 10 (Fig. 2d). Additionally, it resulted in decreased body and liver weight of mice (Fig. 2e, f). Morphological examination and oil red O staining analyses demonstrated that the administration of Ad-PANX1 mitigated liver lipid deposition (Fig. 2g, h), which was further supported by reduced liver TG content (Fig. 2i). While there was no significant effect on serum TG levels following Ad-PANX1 injection (Fig. 2j), it did elevate serum ATP content of *db/db* mice (Fig. 2k, *P* < 0.05). Notably, injecting Ad-PANX1 specifically increased *PANX1* mRNA level in the liver (Fig. 2l) but no other major metabolic tissues including heart, pancreas, muscle, and white adipose tissues (Additional file 1: Fig. S2a). Furthermore, PANX1 overexpression suppressed the mRNA levels of *PEPCK* and *G-6-Pase*, while activating the Akt signaling pathway, reducing FOXO1 expression, and repressing the levels of crucial gluconeogenic proteins in the livers of *db/db* mice (Fig. 2l, m).

**Fig. 3:**
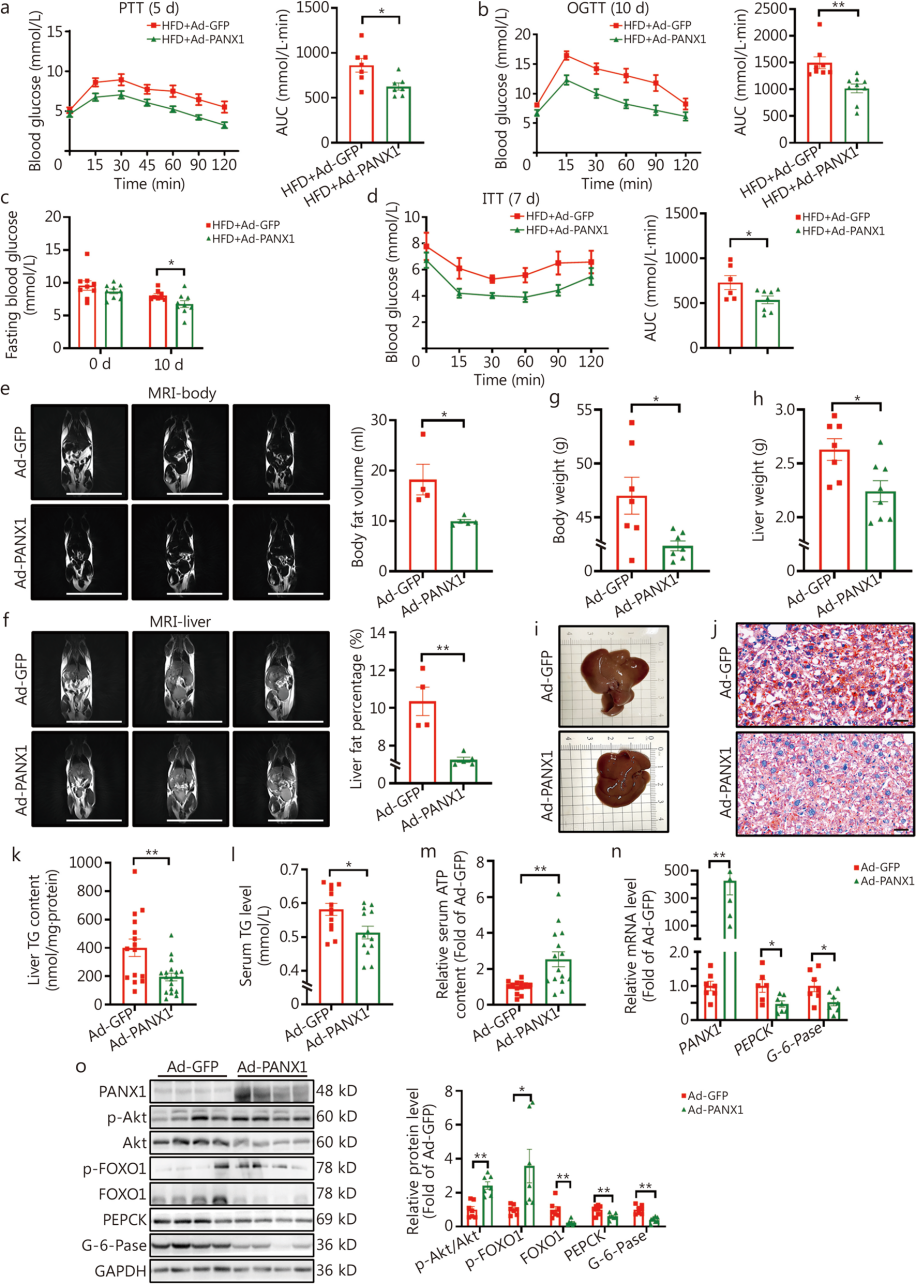
Hepatic pannexin 1 (PANX1) overexpression reduced hyperglycemia and attenuated fatty liver in high-fat diet (HFD)-fed mice. **a** The left panel displayed the pyruvate tolerance test (PTT) curves on day 5 following adenoviral injection in HFD-fed mice, and the right panel presented the corresponding area under the curve (AUC) data (*n* = 7). **b** The left panel displayed the oral glucose tolerance test (OGTT) curves on day 10 post adenoviral injection in HFD-fed mice, and the right panel presented the corresponding AUC data. *n* = 8 (HFD + Ad-GFP) and 9 (HFD + Ad-PANX1). **c** PANX1 overexpression reduced fasting blood glucose level in HFD-fed mice on day 10 post adenoviral injection, from left to right, *n* = 9, 9, 8, and 9, respectively. **d** Liver overexpression of PANX1 increased global insulin sensitivity in HFD-fed mice diet. Another set of HFD-fed mice was used for the insulin tolerance test (ITT). *n* = 6 (HFD + Ad-GFP) and 8 (HFD + Ad-PANX1). The left panel displayed the ITT curves on day 7 post adenoviral injection in HFD-fed mice, and the right panel presented the corresponding AUC data. **e** PANX1 overexpression decreased global fat deposition in HFD-fed mice. The left panel displayed representative magnetic resonance imaging (MRI) images of whole-body fat, while the right panel presented quantitative data, *n* = 4 (Ad-GFP) and 5 (Ad-PANX1). Scale bar = 5 cm. **f** Hepatic PANX1 overexpression reduced hepatic lipid deposition in HFD-fed mice. The left panel displayed representative MRI images of liver fat, while the right panel presented quantitative data, *n* = 4 (Ad-GFP) and 5 (Ad-PANX1). Scale bar = 5 cm. Body weight (**g**, *n* = 7) and liver weight (**h**, from left to right, *n* = 7 and 8) on day 11 post adenoviral injection in HFD mice. Morphological observations (**i**) and oil red O staining analyses (**j**) of livers from Ad-GFP- and Ad-PANX1-injected HFD mice (*n* = 5). Scale bar = 50 μm. Hepatic (**k**, from left to right, *n* = 15 and 18) and serum (**l**, *n* = 13) triglyceride (TG) content in HFD mice after PANX1 overexpression. **m** Overexpression of hepatic PANX1 led to an increase in serum adenosine triphosphate (ATP) content in HFD-fed mice, *n* = 13 (Ad-GFP) and *n* = 15 (Ad-PANX1). **n** Comparative mRNA levels of gluconeogenic genes in HFD-fed mice livers, from left to right, *n* = 7, 7, 6, 7, 7 and 7, respectively. **o** PANX1 overexpression reduced gluconeogenic proteins in HFD-fed mouse livers. The left panel showed representative gel images and the right panel presented quantitative data (*n* = 7). ^*^P < 0.05 or ^**^P < 0.01. HFD + Ad-GFP/Ad-GFP Ad-GFP-injected HFD-fed mice, HFD + Ad-PANX1/Ad-PANX1 Ad-PANX1-injected HFD-fed mice, Akt protein kinase B, FOXO1 forkhead box protein O1, PEPCK phosphoenolpyruvate carboxykinase, G-6-Pase glucose-6-phosphatase

PANX1 was further activated in the livers of HFD mice to confirm its effects on hepatic glucolipid metabolism. Before adenoviral injection, two groups of HFD mice exhibited no difference in OGTT (Additional file 1: Fig. S2b). Ad-PANX1 injection inhibited hepatic glucose production, improved glucose intolerance, decreased fasting hyperglycemia (*P* < 0.05), and enhanced global insulin sensitivity in mice (Fig. 3a-d). MRI analyses demonstrated that Ad-PANX1 injection lowered whole-body and liver fat content (Fig. 3e, f), which was supported by the decrease in body and liver weight (Fig. 3g, h). Ad-PANX1 injection reduced hepatic lipid deposition and serum TG levels in mice (Fig. 3i-l). Furthermore, Ad-PANX1 injection elevated serum ATP content (Fig. 3m, *P* < 0.01). PANX1 overexpression increased *PANX1* mRNA specifically in the liver (Fig. 3n) while showing no effect on other major metabolic tissues including the heart, pancreas, muscle, and white adipose tissues of HFD mice (Additional file 1: Fig. S2c). Moreover, PANX1 overexpression inhibited the mRNA expressions of gluconeogenic genes in the liver of the HFD mouse model (Fig. 3n) and activated the Akt signaling pathway while decreasing the protein levels of FOXO1 and its downstream gluconeogenic genes within the livers of HFD mice (Fig. 3n, o).

To further validate the roles of hepatic PANX1 in glucolipid metabolism, a hepatocyte-specific PANX1 inhibition AAV (AAV-shPANX1) was constructed and verified in hepatocytes (Additional file 1: Fig. S2d). Given that metabolic dysfunction mainly occurred after HFD feeding rather than ND (data not shown), the following experiments focused on the hepatic PANX1 inhibition in HFD mice. Before viral injection, two sets of mice showed no difference in OGTT results (Additional file 1: Fig. S3a). These mice were then injected with AAV-Scramble or AAV-shPANX1, and subjected to HFD feeding. After 8-week HFD feeding, AAV-shPANX1-treated mice exhibited increased hepatic glucose production, glucose intolerance, and insulin resistance compared to control mice (Additional file 1: Fig. S3b-d). MRI analysis revealed an increase in global and liver fat content in mice injected with AAV-shPANX1 (Additional file 1: Fig. S3e, f), supported by elevated liver weight measurements (Additional file 1: Fig. S3g). Morphological and oil red O staining analyses indicated that PANX1 inhibition increased lipid deposition and TG content in mouse livers (Additional file 1: Fig. S3h-j). AAV-shPANX1 injection also elevated serum TG levels with little effect on serum ATP content (Additional file 1: Fig. S3k, l). Hepatic PANX1 inhibition increased the mRNA levels of *PEPCK* and *G-6-Pase* (Additional file 1: Fig. S3m), inactivated Akt, and elevated gluconeogenic proteins in livers (Additional file 1: Fig. S3n).

**Fig. 4:**
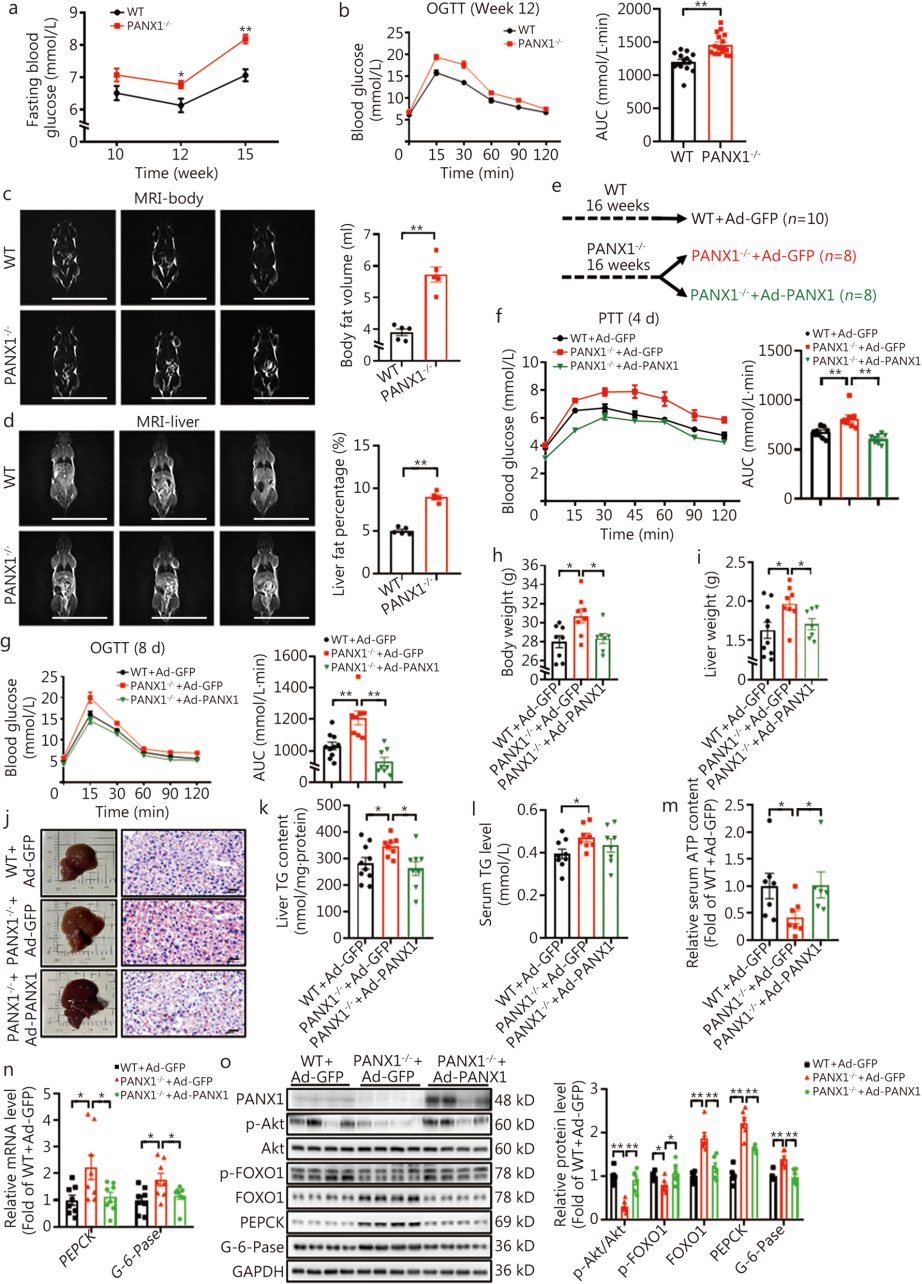
Hepatic pannexin 1 (PANX1) restoration rescued the dysregulated glucolipid metabolism in PANX1-deficient mice. **a** Fasting blood glucose levels of wild-type (WT) and PANX1-deficient mice. The mice were fed on normal chow, from left to right, *n* = 8, 14, and 14 (WT), *n* = 11, 16, and 15 (PANX1^−/−^), respectively. **b** The left panel displayed the glucose tolerance test (OGTT) curves of 12-week-old WT and PANX1-deficient mice, and the right panel presented the area under the curve (AUC) data, *n* = 14 (WT) and 16 (PANX1^−/−^). **c** Body fat was increased in PANX1-deficient mice at the age of 15 weeks old. The left panel displayed the representative magnetic resonance imaging (MRI) images of whole-body fat, and the right panel presented the quantitative data (*n* = 5). Scale bar = 5 cm. **d** Hepatic fat content was increased in PANX1-deficient mice at the age of 15 weeks old. The left panel displayed the representative MRI images of liver fat, and the right panel presented the quantitative data (*n* = 5). Scale bar = 5 cm. **e** Schematic diagram of the grouping mode. 16 PANX1^−/−^ mice were randomly divided into two groups at the age of 16 weeks, and then injected with Ad-GFP and Ad-PANX1, respectively. **f** The left panel displayed the pyruvate tolerance test (PTT) curves on day 4 post adenoviral injection, and the right panel presented AUC data, *n* = 10 (WT + Ad-GFP), 8 (PANX1^−/−^ + Ad-GFP) and 8 (PANX1^−/−^ + Ad-PANX1). **g** The left panel displayed OGTT curves on day 8 post adenoviral injection, and the right panel presented the AUC data, *n* = 10 (WT + Ad-GFP), 8 (PANX1^−/−^ + Ad-GFP), and 8 (PANX1^−/−^ + Ad-PANX1). Body (**h**, from left to right, *n* = 8, 8 and 7) and liver weight (**i,** from left to right, *n* = 10, 8 and 7) on day 9 post adenoviral injection in WT and PANX1-deficient mice. **j** Morphological observations (left panel) and oil red O staining analyses (right panel) of adenoviral-injected-WT and PANX1-deficient mice (*n* = 5). Scale bar = 50 μm. Hepatic (**k**, from left to right, *n* = 10, 8 and 8) and serum (**l**, from left to right, *n* = 9, 8 and 8) triglyceride (TG) levels of adenoviral-injected-WT and PANX1-deficient mice. **m** Hepatic PANX1 restoration rescued the decreased serum adenosine triphosphate (ATP) content in PANX1-deficient mice, *n* = 7 (WT + Ad-GFP), 7 (PANX1^−/−^ + Ad-GFP) and 6 (PANX1^−/−^ + Ad-PANX1). **n** Relative mRNA levels of gluconeogenic genes in livers of adenoviral-injected-WT and PANX1-deficient mice (*n* = 8). **o** Hepatic PANX1 restoration reduced gluconeogenic proteins in PANX1-deficient mouse livers. The left panel displayed representative gel images, and the right panel presented quantitative data, *n* = 6 (WT + Ad-GFP) and 6 (PANX1^−/−^ + Ad-GFP), from left to right, *n* = 6, 6, 6, 5, and 6 (PANX1^−/−^ + Ad-PANX1), respectively. ^*^P < 0.05 or ^**^P < 0.01. WT wild-type, PANX^−/−^ PANX1-deficient mice, WT + Ad-GFP Ad-GFP-injected wild-type mice, PANX^−/−^ + Ad-GFP Ad-GFP-injected PANX1-deficient mice, PANX^−/−^ + Ad-PANX1 Ad-PANX1-injected PANX1-deficient mice, Akt protein kinase B, FOXO1 forkhead box protein O1, PEPCK phosphoenolpyruvate carboxykinase, G-6-Pase glucose-6-phosphatase

Global *PANX1* knockout mice were employed to further consolidate the roles of PANX1 in glucolipid metabolism. The identification of PANX1^−/−^ mice was presented in Additional file 1: Fig. S4a. At 12 weeks old, PANX1-null mice displayed a notable elevation in fasting blood glucose levels compared with wild-type (WT) mice (Fig. 4a). PANX1^−/−^ mice exhibited impaired glucose tolerance, increased hepatic glucose production, and decreased insulin sensitivity at the age of 12 – 15-week-old under ND feeding (Fig. 4b, Additional file 1: Fig. S4b-d). MRI analyses illustrated that PANX1 deficiency led to an augmentation in total body fat volume and liver fat deposition (Fig. 4c, d). To further corroborate the involvement of hepatic PANX1 in the disruption of glucolipid metabolism, the above PANX1^−/−^ mice were classified into two groups, with no significant difference observed in OGTT at the age of 16 weeks. Subsequently, the mice were injected with either Ad-GFP or Ad-PANX1, respectively (Fig. 4e, Additional file 1: Fig. S4e). Increased hepatic glucose production, impaired glucose tolerance, and elevated body and liver weight of PANX1-deficient mice were rescued by Ad-PANX1 injection (Fig. 4f-i, *P* < 0.05). Hepatic restoration of PANX1 reversed the increased liver lipid deposition and TG content without significantly affecting serum TG levels in PANX1-deficient mice (Fig. 4j-l). Serum ATP level was decreased in PANX1-deficient mice but was increased after Ad-PANX1 injection (Fig. 4m, *P* < 0.05). Deficiency of PANX1 resulted in elevated mRNA and protein levels of gluconeogenic genes, which were reversed by PANX1 restoration in mouse livers (Fig. 4n, o). In support, PANX1 deficiency reduced Akt phosphorylation while increasing FOXO1 protein; these effects were subsequently reversed following PANX1 restoration in mouse livers (Fig. 4n, o). Collectively, these findings reveal that hepatic PANX1 plays a critical role in maintaining glucose and lipid homeostasis.

### PANX1 suppressed gluconeogenesis and lipid deposition via ATP-P2 receptor pathway in hepatocytes

**Fig. 5:**
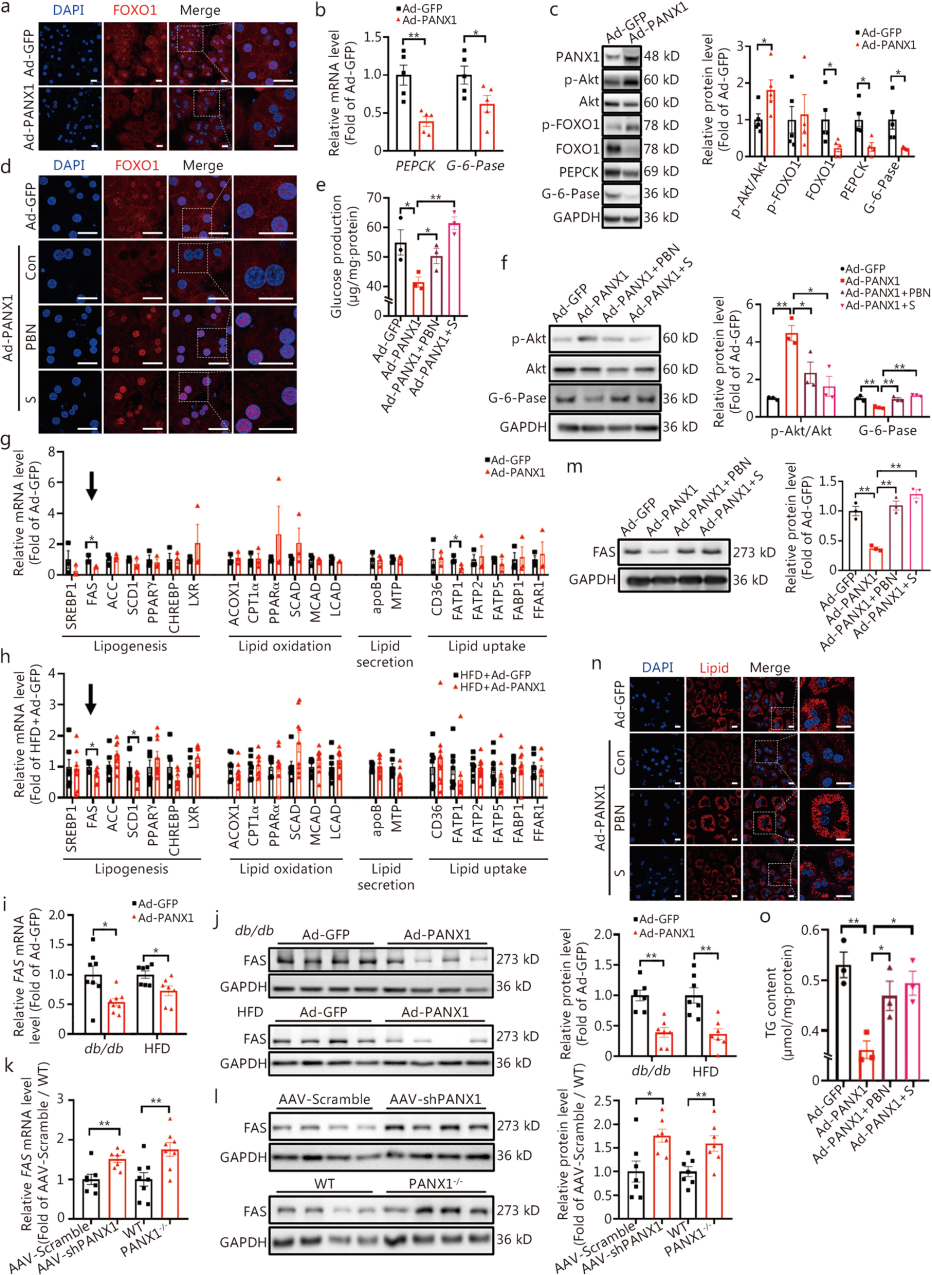
Pannexin 1 (PANX1)-mediated ATP release inhibited gluconeogenesis and reduced lipid deposition by activating P2 receptors in hepatocytes. **a** Representative immunofluorescent staining images of forkhead box protein O1 (FOXO1) in mouse hepatocytes infected with Ad-PANX1 or Ad-GFP (*n* = 3). Scale bar = 25 μm. **b** Relative mRNA levels of gluconeogenic genes in mouse hepatocytes infected with Ad-GFP or Ad-PANX1 (*n* = 5). **c** The left panel displayed representative gel images of gluconeogenic proteins in mouse hepatocytes infected with Ad-GFP or Ad-PANX1, with quantitative data presented in the right panel, *n* = 5 (Ad-GFP), from left to right, *n* = 5, 4, 5, 4 and 5 (Ad-PANX1), respectively. Treatment with PANX1 inhibitor PBN or P2 receptor inhibitor suramin abolished PANX1-induced FOXO1 nuclear exclusion (**d**) and inhibition of glucose production (**e**) in mouse hepatocytes (*n* = 3). Scale bar = 25 μm. **f** Treatment with PBN or suramin abolished PANX1-induced activation of protein kinase B (Akt) in mouse hepatocytes. The left panel displayed the representative gel images, and the right panel presented the quantitative data (*n* = 3). **g** Relative mRNA levels of key lipid metabolic genes in PANX1-overexpressed mouse hepatocytes (*n* = 3). **h** Relative mRNA levels of key lipid metabolic genes in livers of Ad-PANX1-injected HFD mice, *n* = 7 (HFD + Ad-GFP), from left to right, *n* = 8, 7, 9, 9, 9, 9, 9, 9, 9, 9, 9, 9, 9, 9, 8, 9, 9, 9, 9, 9 and 9 (HFD + Ad-PANX1), respectively. **i** PANX1 overexpression reduced the mRNA level of FAS in *db/db* (*n* = 8) and HFD mouse livers (*n* = 7). **j** PANX1 overexpression decreased the fatty acid synthase (FAS) protein level in livers of *db/db* and HFD mice. The left panel displayed representative gel images, and the right panel presented the quantitative data. (*n* = 7). **k** The *FAS* mRNA level was elevated in the livers of *PANX1*-knockdown mice (*n* = 7) and PANX1-deficient mice (*n* = 8). **l** The FAS protein was elevated in the livers of hepatic *PANX1*-knockdown mice and PANX1-deficient mice. The left panel presented the representative gel images, and the right panel displayed the quantitative data (*n* = 7). **m** Treatment with PBN or suramin reversed PANX1-induced inhibition of FAS level in mouse hepatocytes. The left panel presented the representative gel images, and the right panel displayed the quantitative data (*n* = 3). Treatment with PBN or suramin reversed PANX1-induced decrease in lipid accumulation (**n**) and triglyceride (TG) levels (**o**) in mouse hepatocytes (*n* = 3). Scale bar = 25 μm. ^*^P < 0.05 or.^**^P < 0.01. Con control, PBN probenecid, S suramin, DAPI 4,6-diamino-2-phenyl indole, Ad-GFP Ad-GFP-infected mouse hepatocytes, Ad-PANX1 Ad-PANX1-infected mouse hepatocytes, PEPCK phosphoenolpyruvate carboxykinase, G-6-Pase glucose-6-phosphatase, SREBP1 sterol regulatory element-binding protein 1, ACC acetyl-coA carboxylase, SCD1 stearoyl-coA desaturase 1, PPARγ peroxisome proliferator-activated receptor γ, CHREBP carbohydrate response element binding protein, LXR liver x receptor, ACOX1 acyl-CoA oxidase 1, CPT1α carnitine palmitoyltransferase 1α, PPARα peroxisome proliferator-activated receptor α, SCAD short-chain acyl-coA dehydrogenase, MCAD medium-chain acyl-coA dehydrogenase, LCAD long-chain acyl-coA dehydrogenase, apoB apolipoprotein b, MTP microsomal triglyceride transfer protein, CD36 cluster of differentiation 36, FATP1 fatty acid transport protein 1, FATP2 fatty acid transport protein 2, FATP5 fatty acid transport protein 5, FABP1 fatty acid binding protein 1, FFAR1 free fatty acids receptor 1

An intensive study was conducted to pinpoint the mechanism(s) of PANX1 in ameliorating hepatic gluconeogenesis and lipid deposition. Overexpression of PANX1 promoted nuclear exclusion of FOXO1 in mouse and human hepatocytes (Fig. 5a, Additional file 1: Fig. S5a). Furthermore, PANX1 overexpression led to decreased mRNA and protein levels of gluconeogenic genes in both mouse and human hepatocytes (Fig. 5b, c, Additional file 1: Fig. S5b, c). Given that PANX1 facilitated ATP passage, an investigation was undertaken to ascertain whether its positive effects on glucolipid metabolism relied on ATP release and P2 receptors. Firstly, we confirmed that PANX1 inhibitor PBN blocked PANX1-induced ATP release in mouse hepatocytes (Additional file 1: Fig. S5d). Except P2X2, P2X6, and P2Y11, 5 P2X receptors (P2X1, P2X3, P2X4, P2X5, P2X7) and 7 P2Y receptors (P2Y1, P2Y2, P2Y4, P2Y6, P2Y12, P2Y13, P2Y14) were detected by RNA-sequencing in mouse hepatocytes (Additional file 1: Fig. S5e). Administration of either PBN or the P2 receptor pan inhibitor suramin nullified the nuclear exclusion of FOXO1 induced by PANX1 and inhibition of glucose production in mouse (Fig. 5d, e) and human (Additional file 1: Fig. S5f, g) hepatocytes. Consistently with these findings, PANX1-induced increase in Akt phosphorylation and decrease in gluconeogenic gene expression levels were reversed by PBN or suramin treatment (Fig. 5f). Then, to clarify which specific P2 receptor mediated the benefit of PANX1 on inhibiting gluconeogenesis, the pan P2X inhibitor PPADS, the specific P2Y1 inhibitor MRS2179 or the specific P2Y12 inhibitor PSB-0739 was administrated to the PANX1-overexpressed mouse hepatocytes, respectively. Partial inhibition of glucose production induced by PANX1 was observed when using either the P2Y1 or P2Y12 inhibitor, while no significant effect on glucose production was observed with the P2X inhibitor PPADS in mouse hepatocytes (Additional file 1: Fig. S5h). These findings indicate that PANX1 inhibits hepatic gluconeogenesis by releasing ATP and mainly activating the P2Y receptor signaling pathway.

In investigating the mechanism by which PANX1 modulates lipid metabolism, key genes involved in various aspects of lipid metabolism, including lipogenesis, lipid oxidation, lipid secretion, and lipid uptake were detected in PANX1-overexpressed hepatocytes and livers. As a result, *FAS* was the only gene that was consistently downregulated by PANX1 in both hepatocytes (Fig. 5g, *P* < 0.05) and HFD mouse livers (Fig. 5h, *P* < 0.05). The overexpression of PANX1 led to a reduction in FAS protein level in cultured mouse hepatocytes (Additional file 1: Fig. S5i). Meanwhile, the *FAS* mRNA and protein levels were also decreased upon PANX1 overexpression in HepG2 cells (Additional file 1: Fig. S5j, k) as well as mouse livers (Fig. 5i, j). Supporting this finding, the FAS protein level increased when *PANX1* was knocked down or deficient in mouse livers (Fig. 5k, l). PANX1-induced inhibition of FAS protein level and lipid deposition could be reversed by PBN or suramin treatment in mice (Fig. 5m-o) and human hepatocytes (Additional file 1: Fig. S5l, m). Additionally, PANX1-induced inhibition of lipid deposition was partly reversed by P2Y1 and P2Y12 inhibitors, but not by P2X inhibitor PPADS in mouse hepatocytes (Additional file 1: Fig. S5n).

### PANX1 inhibited the JNK-AP1-FAS pathway to ameliorate hepatic lipid deposition

**Fig. 6:**
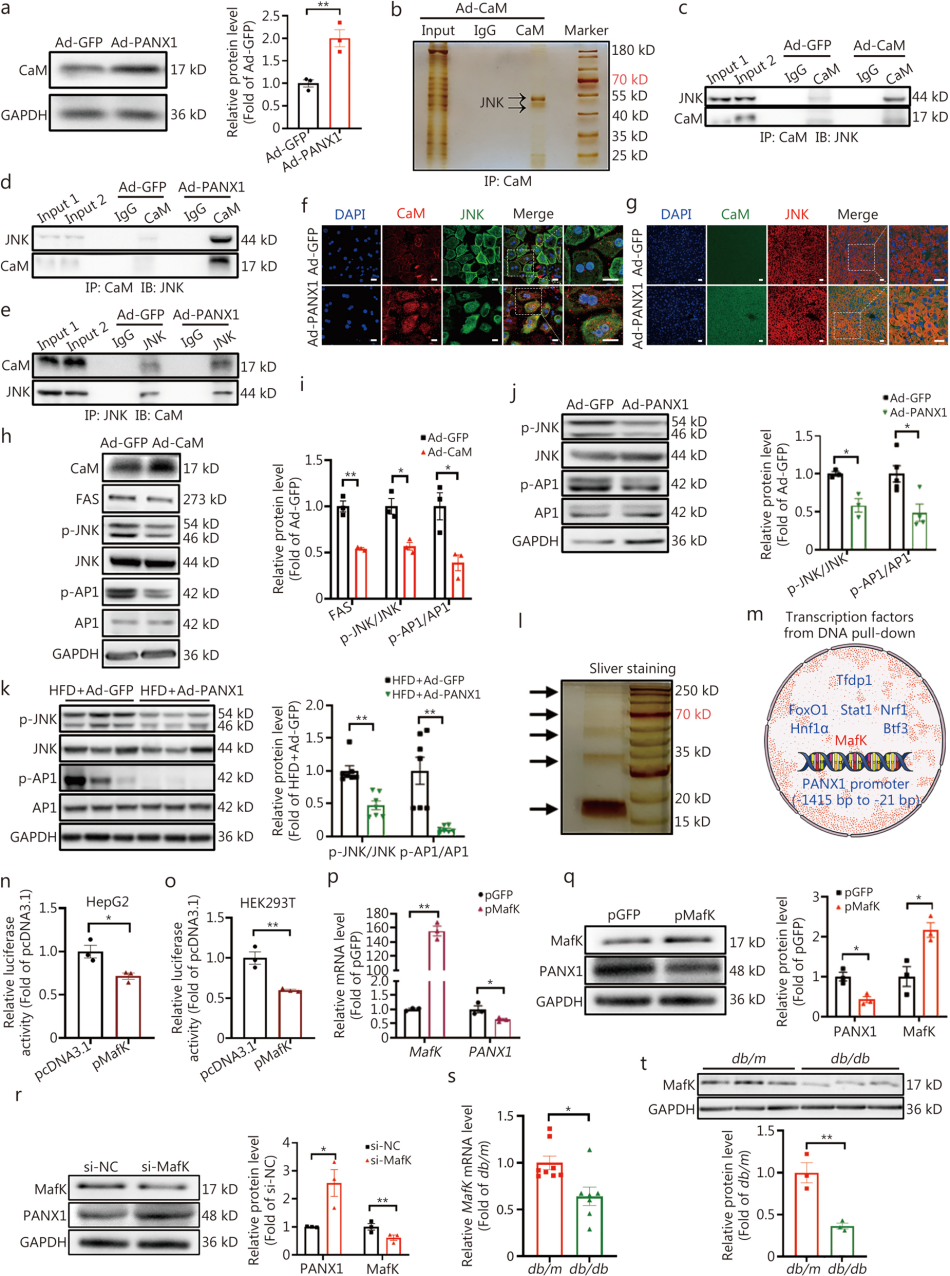
Pannexin 1 (PANX1) inhibited c-Jun N-terminal kinase-activator protein-1-fatty acid synthase (JNK-AP1-FAS) pathway to ameliorate hepatic lipid deposition. **a** PANX1 overexpression elevated calmodulin (CaM) protein level in mouse hepatocytes. The left panel showed the representative gel images, while the right panel displayed quantitative data (*n* = 3). **b** Representative silver staining gel image of Co-IP products in Ad-CaM-treated mouse hepatocytes. The bands indicated by the arrows were pooled and analyzed by mass spectrometry (MS). Overexpression of CaM (**c**) or PANX1 (**d** and **e**) stimulated CaM-JNK interaction in mouse hepatocytes (*n* = 3). **f** Representative immunofluorescent staining images of CaM and JNK in Ad-GFP- or Ad-PANX1-overexpressed mouse hepatocytes (*n* = 3). Scale bar = 25 μm. **g** Representative immunofluorescent staining images of CaM and JNK in liver sections of HFD-fed mice injected with Ad-GFP or Ad-PANX1 (*n* = 3). Scale bar = 25 μm. **h** and **i** CaM overexpression decreased the phosphorylations of JNK and AP1 in mouse hepatocytes. Panel (**h**) presented the representative gel images, and panel (**i**) displayed the quantitative data (*n* = 3). PANX1 overexpression decreased JNK and AP1 phosphorylations in mouse hepatocytes (**j,** from left to right, *n* = 3, 3, 5, and 4, respectively) and HFD-fed mouse livers (**k**, *n* = 7). The left panel displayed the representative gel images, and the right panel presented the quantitative data. **l** Representative silver staining gel image of DNA-pulldown products in mouse hepatocytes. The bands indicated by the arrows were subjected to MS analysis. **m** Schematic diagram of nuclear proteins identified by MS analysis. V-maf musculoaponeurotic fibrosarcoma oncogene homolog K (MafK) overexpression inhibited the transcriptional activity of *PANX1* gene promoter in HepG2 (**n**) and HEK 293 T (**o**) cells (*n* = 3). **p** MafK overexpression reduced the mRNA level of *PANX1* in mouse hepatocytes (*n* = 3). **q** Overexpression of MafK decreased PANX1 protein level in mouse hepatocytes. The left panel showed representative gel images and the right panel displayed quantitative data (*n* = 3). **r** siRNA silencing of MafK elevated PANX1 protein level in mouse hepatocytes. The left panel showed representative gel images and the right panel presented quantitative data (*n* = 3). **s** The mRNA level of *MafK* was decreased in the livers of *db/db* mice, *n* = 8 (*db/m*) and 7 (*db/db*). **t** The protein level of MafK was reduced in livers of *db/db* mice. The upper panel showed representative gel images and the lower panel displayed quantitative data (*n* = 3). ^*^P < 0.05 or ^**^P < 0.01. Ad-GFP Ad-GFP-infected mouse hepatocytes, Ad-PANX1 Ad-PANX1-infected mouse hepatocytes, Ad-CaM Ad-CaM-treated mouse hepatocytes, FAS fatty acid synthase, Tfdp1 transcription factor dp 1, FoxO1 forkhead box protein O1, Stat1 signal transducer and activator of transcription 1, Nrf1 nuclear respiratory factor 1, Hnf1α hepatocyte nuclear factor 1α, Btf3 basic transcription factor 3, p-JNK phosphorylated c-Jun N-terminal kinase, p-AP1 phosphorylated activator protein-1, DAPI 4,6-diamino-2-phenyl indole, IP immunoprecipitation, pcDNA3.1 vector plasmid as control, pGFP GFP plasmid transfection, pMafK MafK plasmid transfection, si-NC control siRNA transfection, si-MafK MafK siRNA transfection

Previously, we had demonstrated that increased ATP release acted on P2 receptors to activate CaM [16, 22, 25] in hepatocytes. The protein level of CaM was elevated in hepatocytes following PANX1 overexpression (Fig. 6a, *P* < 0.01). Upon activation, CaM directly interacted with multiple intracellular proteins to regulate gene expression in hepatocytes and pancreatic β cells, respectively [19, 22]. Therefore, Co-IP combined with MS was employed to uncover potential proteins interacting with CaM in PANX1-overexpressed hepatocytes. As a result, JNK, also known as mitogen-activated protein kinase 9, was identified as one of the potential CaM-interacting proteins in hepatocytes (Fig. 6b, Additional file 1: Table S4). Further studies confirmed the interaction between CaM and JNK, which was augmented after CaM (Fig. 6c) or PANX1 (Fig. 6d, e) overexpression in hepatocytes. Immunofluorescent staining revealed the increased co-localization of CaM and JNK in PANX1-overexpressed hepatocytes and mouse livers (Fig. 6f, g). A previous study has demonstrated that phosphorylated JNK can phosphorylate and activate the transcription factor AP1 [43, 44], which could upregulate the protein level of FAS [45]. In this current investigation, we found that the phosphorylation of JNK and AP1 was suppressed by CaM overexpression in mouse hepatocytes (Fig. 6h, i). Subsequent dual-luciferase reporter gene detection revealed that CaM overexpression blunted AP1-promoted activation of the *FAS* gene promoter (Additional file 1: Fig. S6a). Consistently, PANX1 overexpression also inhibited the phosphorylations of JNK and AP1 in mouse hepatocytes (Fig. 6j) and HFD-fed mouse livers (Fig. 6k). Taken together, PANX1-mediated ATP release activated CaM via P2 receptor to interact with JNK while inhibiting its activity, dephosphorylating and inactivating the transcription factor AP1 to repress FAS protein level and lipid synthesis in hepatocytes.

### FFAs upregulated PANX1 through MafK

As previously determined, the upregulation of hepatic PANX1 serves as a compensatory mechanism against metabolic disorders. The potential mechanism behind the induction of PANX1 upregulation by FFAs was further investigated. A DNA probe was used to pull-down the potential transcriptional factors that bind to the promoter region (-1415 to -21 bp) of the mouse *PANX1* gene. Subsequent MS analysis identified several potential nuclear proteins including hepatocyte nuclear factor 1α (Hnf1α), signal transducer and activator of transcription (Stat1), nuclear respiratory factor 1 (Nrf1), transcription factor Dp-1 (Tfdp1), basic transcription factor 3 (Btf3), FoxO1, and v-maf musculoaponeurotic fibrosarcoma oncogene homolog K (MafK) in the DNA-pull-down products indicated by the arrows (Fig. 6l, m, Additional file 1: Table S5). The transcription factor MafK belonged to the v-Maf transcription factor family, and bioinformatic prediction revealed a specific binding site for v-Maf in the mouse *PANX1* gene promoter (Additional file 1: Fig. S6b). In contrast, no potential binding sites for Hnf1α, Stat1, Nrf1, and FoxO1 were found in the promoter region of the mouse *PANX1* gene. Therefore, whether MafK was involved in FFAs-induced upregulation of PANX1 expression in hepatocytes was probed. Luciferase reporter analyses revealed that MafK overexpression inhibited the transcriptional activity of PANX1 in HepG2 (*P* < 0.05) and HEK 293 T (*P* < 0.01) cells (Fig. 6n, o). Transfection with MafK plasmid reduced the mRNA levels of *PANX1* in mouse and human hepatocytes (Fig. 6p, Additional file 2: Fig. S6c). Meanwhile, the protein level of PANX1 also decreased in mouse hepatocytes induced by MafK overexpression (Fig. 6q). Silencing *MafK* using siRNA increased both mRNA (Additional file 1: Fig. S6d) and protein (Fig. 6r) levels of *PANX1* in mouse hepatocytes. Furthermore, the *MafK* mRNA and protein levels were decreased in the livers of *db/db* (Fig. 6s, t) and HFD mice (Additional file 1: Fig. S6e, f). These findings indicate that MafK is a negative regulator of *PANX1* gene transcription. Under the obese condition, fatty acids repressed MafK expression to increase PANX1 transcription.

### FAM3A-mediated ATP release was dependent on PANX1

**Fig. 7:**
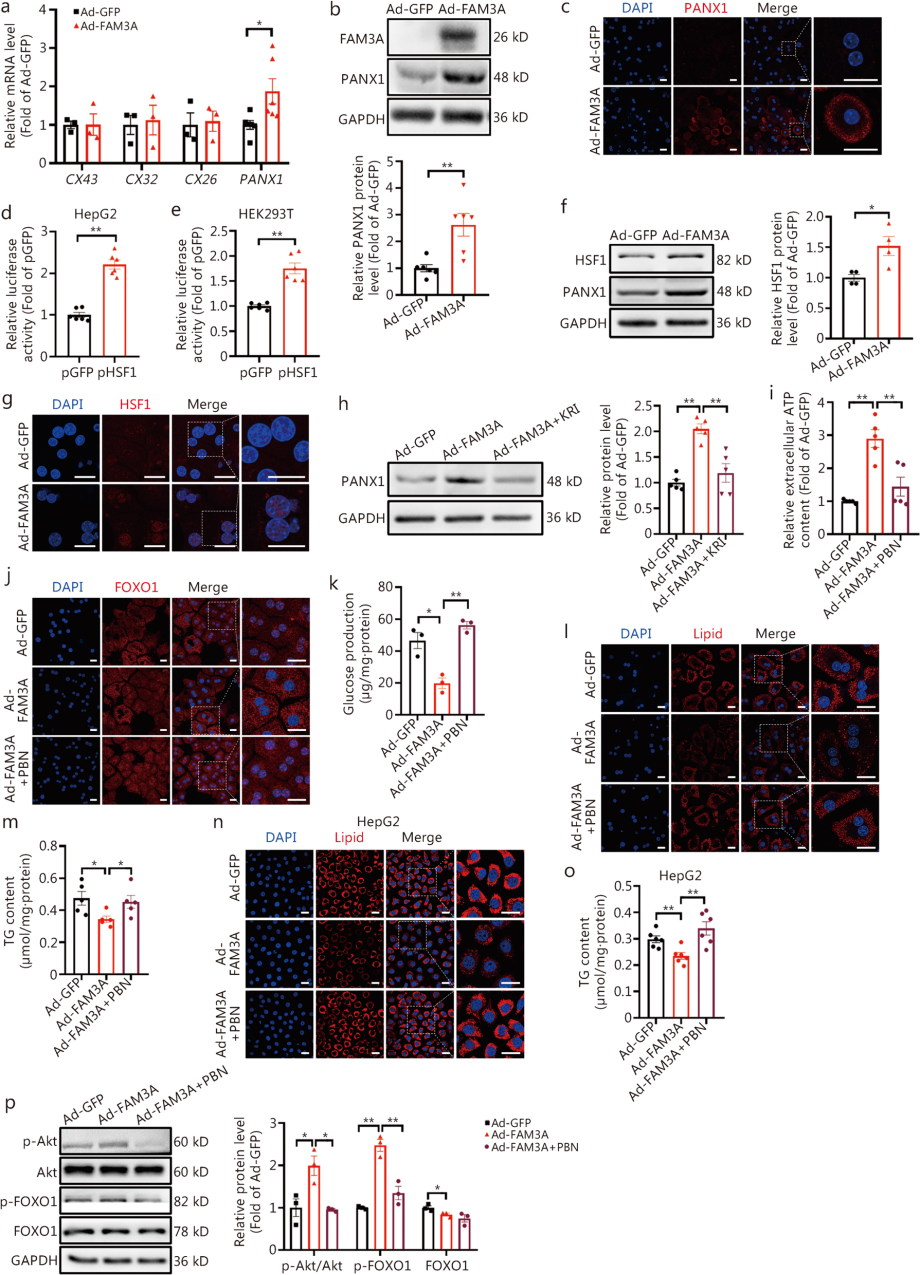
Family with sequence similarity 3 member A (FAM3A)-mediated ATP release was dependent on pannexin 1 (PANX1). **a** Overexpression of FAM3A elevated *PANX1* mRNA level in mouse hepatocytes, from left to right, *n* = 3, 3, 3, 3, 3, 3, 6, and 6, respectively. **b** Overexpression of FAM3A upregulated PANX1 protein level in mouse hepatocytes. The upper panel showed representative gel images, and the lower panel displayed the quantitative data (*n* = 6). **c** Representative immunofluorescent staining images of PANX1 in FAM3A-overexpressed mouse hepatocytes (*n* = 3). Scale bar = 25 μm. Heat shock factor 1 (HSF1) overexpression enhanced the transcriptional activity of the *PANX1* gene promoter in HepG2 (**d**) and HEK 293 T (**e**) cells (*n* = 6). **f** Overexpression of HSF1 upregulated PANX1 protein level in mouse hepatocytes. The left panel showed representative gel images and the right panel displayed quantitative data (*n* = 4). **g** Representative immunofluorescent staining images of HSF1 in FAM3A-overexpressed mouse hepatocytes (*n* = 3). Scale bar = 25 μm. **h** Treatment with HSF1 inhibitor abolished FAM3A-induced increase in PANX1 protein level in mouse hepatocytes. The left panel showed representative gel images and the right panel displayed quantitative data (*n* = 5). **i** Pannexin 1 inhibitor PBN treatment blocked FAM3A-induced ATP release in mouse hepatocytes (*n* = 5). PBN treatment reversed FAM3A-promoted FOXO1 nuclear exclusion (**j**) and glucose production suppression (**k**) in mouse hepatocytes (*n* = 3). Scale bar = 25 μm. PBN treatment reversed FAM3A-induced reduction in lipid accumulation (**l**, *n* = 3) and triglyceride (TG) levels (**m**, *n* = 5) in mouse hepatocytes. Scale bar = 25 μm. PBN treatment reversed FAM3A-induced reduction in lipid deposition (**n**, *n* = 3) and TG content (**o**, *n* = 6) in HepG2 cells. Scale bar = 25 μm. **p** PBN treatment blocked FAM3A-induced Akt and FOXO1 phosphorylations in mouse hepatocytes. The left panel showed representative gel images and the right panel presented quantitative data (*n* = 3). ^*^P < 0.05 or.^**^P < 0.01. KRI HSF1 inhibitor KRIBB11, CXs connexins, Ad-GFP Ad-GFP-infected mouse hepatocytes, Ad-FAM3A Ad-FAM3A-infected mouse hepatocytes, Akt protein kinase B, FOXO1 forkhead box protein O1

We had previously established that FAM3A-induced ATP release is crucial for maintaining hepatic glucose and lipid homeostasis [16, 22, 25]. However, the mechanism by which it promotes ATP release in hepatocytes remained unrevealed. FAM3A overexpression upregulated the *PANX1* mRNA level but not *CX43*, *CX32*, and *CX26* in mice (Fig. 7a) and human hepatocytes (Additional file 1: Fig. S7a). The overexpression of FAM3A also increased the PANX1 protein in mouse (Fig. 7b, c, *P* < 0.01) and human (Additional file 1: Fig. S7b) hepatocytes. To determine the mechanism of FAM3A-promoted PANX1 transcription, the potential transcription factor sites in the promoter regions of human and mouse *PANX1* gene promoters were predicted by bioinformatic methods. As a result, paired box 4 (Pax-4), HSF1, and v-Maf binding sites were identified on the promoter regions of both mouse and human *PANX1* genes (Additional file 1: Fig. S6b, Additional file 1: Fig. S8a). Reference mining revealed that to date, HSF1 but no other transcription factors were reported to participate in the regulation of hepatic glucolipid metabolism [46–48]. HSF1 overexpression activated the transcriptional activity of PANX1 in HepG2 and HEK 293 T cells (Fig. 7d, e, *P* < 0.01), and increased PANX1 protein level in HepG2 and L02 cells (Additional file 1: Fig. S8b, c). Overexpression of FAM3A elevated the protein level of HSF1 in both mouse (Fig. 7f) and human (Additional file 1: Fig. S8d) hepatocytes. Immunofluorescent staining confirmed an increase in nuclear distribution of HSF1 in FAM3A-overexpressed mouse hepatocytes (Fig. 7g). Treatment with HSF1 inhibitor KRIBB11 abolished FAM3A-induced increase in PANX1 protein level in mouse (Fig. 7h) and human hepatocytes (Additional file 1: Fig. S8e).

Furthermore, the dependence of FAM3A-induced ATP release on PANX1 was determined. Treatment with the PANX1 inhibitor PBN effectively blocked FAM3A-induced ATP release in mouse (Fig. 7i) and human (Additional file 1: Fig. S9a) hepatocytes. Additionally, PBN treatment also inhibited FAM3A-induced nuclear exclusion of FOXO1 and inhibition of glucose production in mouse (Fig. 7j, k) and human hepatocytes (Additional file 1: Fig. S9b, c). Meanwhile, PANX1 inhibition also abolished the effects of FAM3A-induced reduction in lipid accumulation in mouse (Fig. 7l, m) and human (Fig. 7n, o) hepatocytes. Consistently, FAM3A-induced phosphorylations of Akt and FOXO1 were also reversed by PBN treatment in mouse hepatocytes (Fig. 7p).

**Fig. 8:**
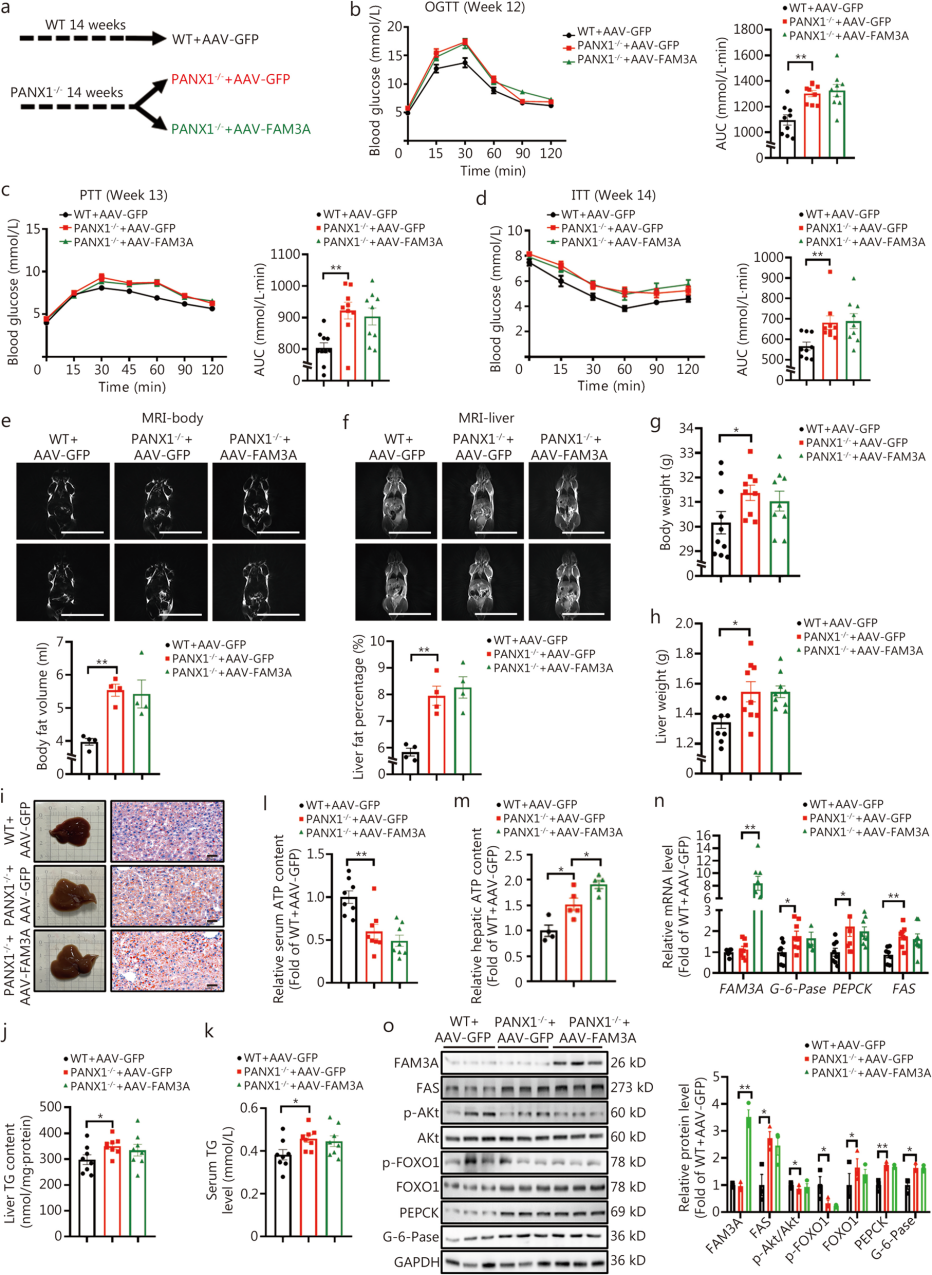
Hepatic family with sequence similarity 3 member A (FAM3A) overexpression failed to rescue the metabolic disorders of pannexin 1 (PANX1)-deficient mice. **a** Schematic diagram of the grouping mode. PANX1^−/−^ mice were randomly divided into two groups at the age of 14 weeks, and then injected with AAV-GFP or AAV-FAM3A, respectively. The mice were fed on normal chow. **b** The left panel showed oral glucose tolerance test (OGTT) curves on week 12 post AAV injection wild-type (WT) and PANX1-deficient mice, and the right panel displayed an area under the curve (AUC) data (*n* = 9). **c** The left panel showed pyruvate tolerance test (PTT) curves on week 13 post-AAV injection in WT and PANX1-deficient mice, and the right panel presented AUC data, *n* = 10 (WT + AAV-GFP), 9 (PANX1^−/−^ + AAV-GFP) and 9 (PANX1^−/−^ + AAV-FAM3A). **d** The left panel displayed insulin tolerance test (ITT) curves on week 14 post-AAV injection in WT and PANX1-deficient mice, and the right panel presented AUC data (*n* = 9). The upper panel showed representative magnetic resonance imaging (MRI) images of whole-body (**e**) and hepatic (**f**) fat in AAV-injected WT and PANX1-deficient mice, and the lower panel displayed quantitative data (*n* = 4). Scale bar = 5 cm. Body (**g**, from left to right, *n* = 10, 9 and 9) and liver weight (**h**, *n* = 9) on the sacrificed day. **i** Morphological observations (left panel) and oil red O staining analyses (right panel) of AAV-WT and PANX1-deficient mice (*n* = 5). Scale bar = 50 μm. Liver (**j**) and serum (**k**) triglyceride (TG) content of AAV-injected WT and PANX1-deficient mice (*n* = 8). **l** Hepatic FAM3A overexpression failed to rescue the decreased serum adenosine triphosphate (ATP) content in PANX1-deficient mice (*n* = 8). **m** FAM3A overexpression elevated ATP level in livers of PANX1-deficient mouse, *n* = 4 (WT + AAV-GFP), 5 (PANX1^−/−^ + AAV-GFP) and 5 (PANX1^−/−^ + AAV-FAM3A). **n** Relative mRNA levels of metabolic genes in livers of mice, from left to right, *n* = 7, 8, 8 and 7 (WT + AAV-GFP), *n* = 8 (PANX1^−/−^ + AAV-GFP) and 8 (PANX1^−/−^ + AAV-FAM3A). **o** The left panel showed representative gel images of metabolic proteins in mouse livers, and the right panel presented quantitative data (*n* = 3). ^*^P < 0.05 or ^**^P < 0.01. WT + AAV-GFP AAV-GFP-injected wild-type mice, PANX^−/−^ + AAV-GFP AAV-GFP-injected PANX1-deficient mice, PANX^−/−^ + AAV-FAM3A AAV-FAM3A-injected PANX1-deficient mice, FAS fatty acid synthase, Akt protein kinase B, FOXO1 forkhead box protein O1, PEPCK phosphoenolpyruvate carboxykinase, G-6-Pase glucose-6-phosphatase

**Fig. 9:**
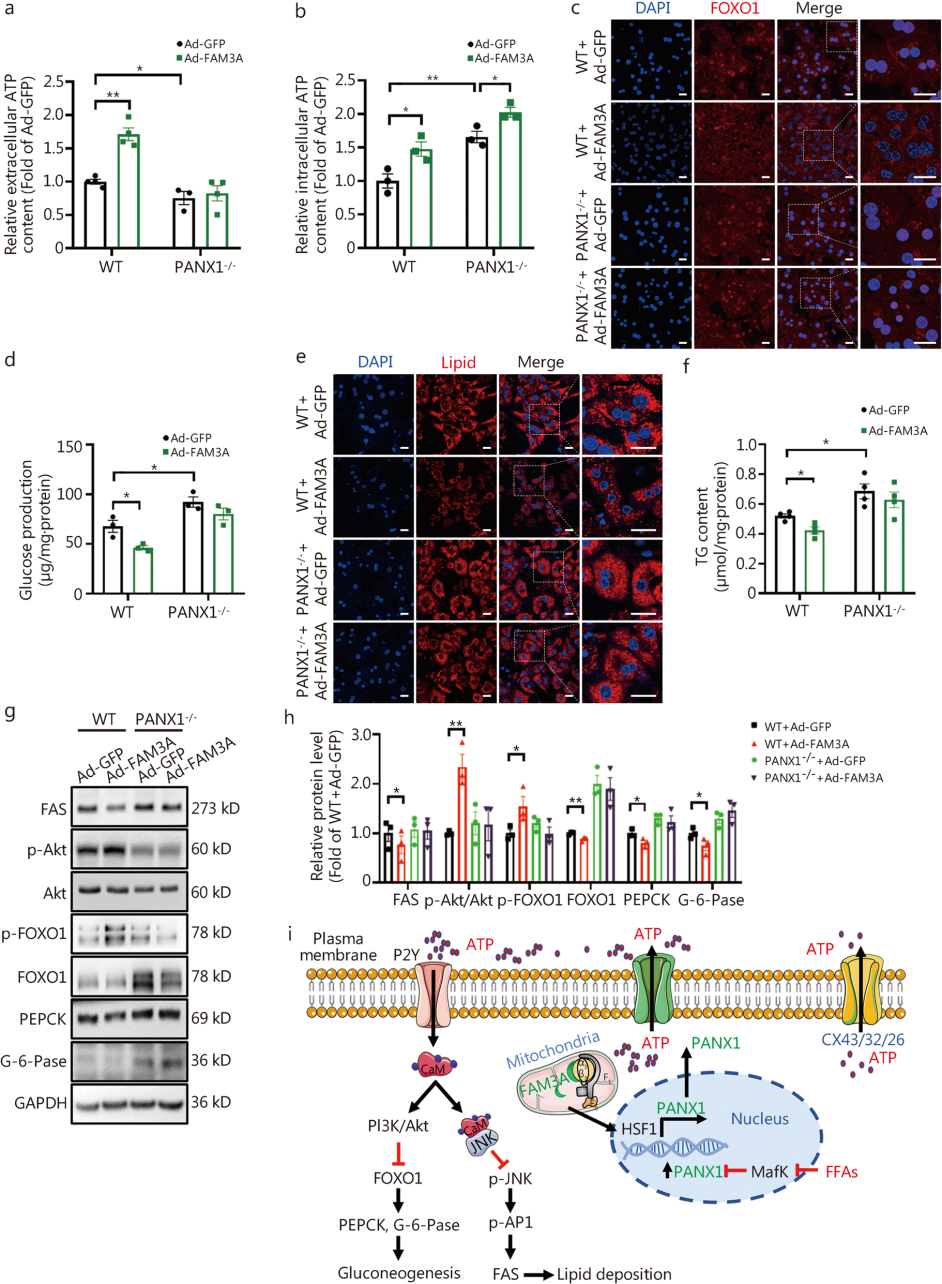
Family with sequence similarity 3 member A (FAM3A) overexpression had no effect on the expressions of metabolic genes in pannexin 1 (PANX1)-deficient hepatocytes. **a** FAM3A overexpression had little effect on the extracellular adenosine triphosphate (ATP) content in PANX1-deficient hepatocytes, from left to right, *n* = 4, 4, 3, and 4, respectively. **b** FAM3A overexpression enhanced intracellular ATP content in PANX1-deficient hepatocytes (*n* = 3). **c** Representative immunofluorescent staining images of forkhead box protein O1 (FOXO1) in PANX1-deficient hepatocytes with FAM3A overexpression (*n* = 3). Scale bar = 25 μm. **d** FAM3A overexpression had little effect on suppressing glucose production in PANX1-deficient hepatocytes (*n* = 3). **e** and **f** FAM3A overexpression failed to reduce lipid accumulation in PANX1-deficient hepatocytes. Panel (**e**) displayed representative lipid confocal images (*n* = 3), and panel (**f**) presented quantitative triglyceride (TG) analysis (*n* = 4). Scale bar = 25 μm. **g** and **h** FAM3A overexpression failed to activate the protein kinase B (Akt) pathway in PANX1-deficient mouse hepatocytes. Panel (**g**) presented representative gel images, and panel (**h**) displayed quantitative data. (*n* = 3). ^*^P < 0.05 or ^**^P < 0.01. **i** Proposed a model of PANX1-mediated ATP release that confers FAM3A’s suppression effects on hepatic gluconeogenesis and lipogenesis. PANX1-mediated ATP release activates purinergic P2 receptors in hepatocytes. On one hand, the activated Akt inhibits FOXO1 from repressing the expressions of PEPCK and G-6-Pase, thereby suppressing gluconeogenesis. On the other hand, activated CaM interacted with JNK to inhibit its activity, thereby repressing AP1 activity and FAS expression to reduce lipid deposition. Under obese conditions, FFAs upregulate PANX1 expression by inhibiting the expressions of the transcription factors MafK. Upregulation of hepatic PANX1 expression is an important compensatory mechanism for maintaining glucose and lipid homeostasis under obese conditions. FAM3A activates HSF1 to induce HSF1 expression, which in return confers its effects on ATP release induction and gluconeogenesis and lipogenesis repression in hepatocytes. Activation of the insulin-independent FAM3A-HSF1-PANX1-ATP-P2 receptor signaling pathway is effective in treating metabolic disorders. Akt protein kinase B, ATP adenosine triphosphate, CaM calmodulin, CX connexin, FAM3A family with sequence similarity 3 member A, FAS fatty acid synthase, FOXO1 forkhead box protein O1, G-6-Pase glucose-6-phosphatase, HSF1 heat shock factor 1, MafK v-maf musculoaponeurotic fibrosarcoma oncogene homolog K, P2R P2 receptor, PANX1 pannexin 1, p-AP1 phosphorylated activator protein-1, PI3K phosphoinositide 3-kinase, p-JNK phosphorylated c-Jun N-terminal kinase, FFAs free fatty acids, WT wild-type

To further investigate whether the regulatory effects of FAM3A on hepatic glucose and lipid depend on PANX1 in vivo, AAV-FAM3A was injected into the tail vein of PANX1-deficient mice, using AAV-GFP as a control. There was no difference observed in OGTT between the two groups of mice prior to AAV injection (Fig. 8a, Additional file 1: Fig. S10a). Notably, long-term hepatic FAM3A overexpression failed to improve the impaired glucose tolerance and elevated fasting hyperglycemia in PANX1-deficient mice (Fig. 8b, Additional file 1: Fig. S10b, c). Increased glucose production and insulin resistance in PANX1-deficient mice were not affected by AAV-FAM3A injection (Fig. 8c, d). MRI revealed that long-term overexpression of hepatic FAM3A had no effect on elevated global body and liver fat content, and body and liver weight (Fig. 8e-h). FAM3A overexpression failed to reduce hepatic lipid deposition as evidenced by morphological observation and TG content measurement in PANX1-deficient mice (Fig. 8i-k). Moreover, although long-term overexpression of hepatic FAM3A increased hepatic ATP levels with little effect on serum ATP content in PANX1-deficient mice (Fig. 8l, m, *P* < 0.05), it failed to activate the Akt signaling pathway and inhibit the expressions of gluconeogenic and lipogenic genes in livers from PANX1^−/−^ mouse (Fig. 8n, o). In cultured hepatocytes lacking PANX1 expression, FAM3A overexpression led to an elevation in intracellular ATP content but did not promote ATP release into extracellular space (Fig. 9a, b). These hepatocytes exhibited excessive glucose production, while enhanced lipid accumulation was observed after FFAs treatment (Fig. 9c-f). The overexpression of FAM3A prompted nuclear exclusion of FOXO1, inhibited glucose production, and decreased lipid accumulation in WT mouse hepatocytes; however, these effects were not observed in PANX1-deficient hepatocytes (Fig. 9c-f). The inhibitory effects of FAM3A on gluconeogenic and lipogenic gene expressions were also nullified in PANX1^−/−^ mouse hepatocytes (Fig. 9g, h). It is evident that PANX1-mediated ATP release confers FAM3A’s regulatory effects on hepatic glucolipid metabolism.

## Discussion

ATP released from cells via various mechanisms functions as a signal molecule to activate P2 receptors in a paracrine or autocrine manner [49]. While cellular ATP release is predominantly mediated by exocytosis and channel proteins in most cell types, there is ongoing debate in different cell types [32]. PANX1, a hemichannel protein, facilitates the ATP release across diverse cell types [29]. In this study, a notable elevation in PANX1 expression was observed in the livers of individuals with NAFLD and diabetic animals. Previous studies have also reported that *PANX1* mRNA was increased in the livers of mice with non-alcoholic steatohepatitis [50] and various other liver diseases [51]. Despite these findings, the specific role and mechanism of hepatic PANX1 in the regulation of glucolipid metabolism remained to be revealed. In the current study, we found that hepatic PANX1 overexpression markedly improved the disorganized glucolipid metabolism of obese mice. Conversely, mice with hepatic *PANX1* knockdown or global *PANX1* knockout exhibited glucolipid metabolic disorders. Our results provided strong evidence for the importance of PANX1-mediated ATP release in maintaining hepatic glucose and lipid homeostasis. Although several studies have demonstrated that the pathological level of extracellular ATP released by apoptotic cells, inflammatory cells or carcinoma cells is a deleterious factor for various biological processes [52, 53], it should be noted that the action of extracellular ATP is concentration-dependent and time-dependent [54]. Generally, nmol/L to low μmol/L levels of extracellular ATP were beneficial for some physiological processes, while high μmol/L to mmol/L levels impaired cell functions [52]. For instance, treatment with ATP at the range of 10 to 50 μmol/L exhibits a synergistic effect with insulin in stimulating the uptake of glucose in skeletal muscle [55]. Another study observed that 50% of glucose uptake is mediated by PANX1 hemichannels through an insulin-independent mechanism in isolated myofibers [56]. Nevertheless, chronic incubation with 1 mmol/L ATP inhibits insulin-stimulated glucose uptake in adipocytes [57]. Palmitic acid stimulated healthy hepatocytes to release ATP by more than fivefold [58]. In a series of previous studies, we have established that the physiological level of extracellular ATP is beneficial and important for glucose and lipid metabolism in various cell types, particularly hepatocytes [16–23, 25]. In the current and previous studies, we consistently observed a modest increase in extracellular ATP levels up to twofold in medium and serum in case of FAM3A or PANX1 overexpression and activation. Our findings strongly propose that tightly controlled ATP release from hepatocytes plays a crucial role in maintaining glucolipid homeostasis.

There is increasing evidence that PANX1 serves as a critical ATP release channel [59, 60]. PANX1 can be activated reversibly by various stimuli such as intracellular Ca^2+^ levels, cell depolarization, mechanical stimulation, and hypoxia [32]. In addition, several other studies have also suggested that PANX1 can be activated by different P2 receptors including P2X4, P2X7, P2Y1, P2Y2, and P2Y6 [59]. In contrast, CXs cannot be activated by P2 receptors despite their structural similarities to PANXs [32]. Under pathological conditions, ATP release mediated by CXs primarily occurs during ischemia–reperfusion injury or inflammatory processes [32]. Activation of insulin receptors can promote PANX1-mediated ATP release, which in turn enhances glucose uptake in adipocytes. Adipocyte-specific *PANX1* knockout exacerbated HFD-induced global insulin resistance [61]. Moreover, PANX1-induced ATP release regulates lipolysis and macrophage migration in adipocytes and modulates adipose stromal cell differentiation and fat accumulation [62, 63]. PANX1 activation also promotes thermogenesis in brown adipose tissue [64]. Consistent with these findings, our previous findings revealed that FAM3A-induced ATP release activated uncoupling protein 1 (UCP1) to promote thermogenesis in brown adipose tissue [22]. However, some studies have also shown that PANX1 deficiency protects mice from acute and chronic liver failure as well as chemically-induced liver fibrosis [50, 51], likely due to a reduction in the release of pathological levels of ATP from apoptotic or injured hepatocytes. The current investigation reveals the crucial roles played by physiological levels of PANX1-mediated ATP release in maintaining hepatic glucolipid homeostasis.

It had been previously established that hepatocyte-released ATP triggers the activation of P2R, subsequently leading to the upregulation and activation of CaM [16, 22, 25]. Several studies have shown that CaM can translocate from the cytoplasm to the nucleus, where it functions as a co-activator of certain transcription factors [19, 22]. For instance, in hepatocytes, nuclear CaM interacts with forehead box protein A2 (FOXA2) to induce the expression of carnitine palmitoyltransferase 2, a pivotal enzyme in regulating fatty acid oxidation [22]. Similarly, CaM also interacts with FOXA2 to upregulate the expression of pancreatic and duodenal homeobox 1 in pancreatic β cells [19]. Here, we further unveiled that PANX1 also stimulated CaM to interact with JNK and inhibit its activity. Inhibition of JNK phosphorylation inactivated the transcription factor AP1, resulting in a decrease in FAS expression and a subsequent reduction in lipid synthesis and deposition. In high uric acid-induced hepatocytes, activated JNK phosphorylates AP1, increasing the transcriptional activity of FAS thus deteriorating hepatic fat accumulation [45]. Additionally, the beneficial effect of PANX1 on lipid deposition was abolished by inhibitors targeting PANX1 or P2 receptors. These findings have highlighted the critical role of PANX1-mediated ATP release in inhibiting lipogenesis in hepatocytes. Although hepatic mRNA expressions of *CX26*, *CX32,* and *CX43* remain unchanged during the development of NAFLD and diabetes, they may still play significant roles in maintaining metabolic homeostasis by providing constitutive ATP release. It is worth investigating their roles in the regulation of hepatic glucolipid metabolism.

HSF1 is a transcription factor that is involved in promoting insulin secretion in pancreatic islet cells and enhancing glucose utilization in skeletal muscle [46, 47]. It has also been shown to protect mice from HFD-induced insulin resistance, hepatic lipid deposition, and obesity in adipose tissues [65]. We had also previously revealed that FAM3C upregulated HSF1 to activate the CaM-PI3K-Akt pathway, thereby suppressing gluconeogenesis in hepatocytes [41]. Additionally, angiotensin II activates HSF1 to stimulate the FAM3A-ATP-P2 receptor pathway, promoting the phenotypic switching of vascular smooth muscle cells [20]. Here, we further demonstrated that FAM3A promoted the transcription of PANX1 through HSF1. Blockade of HSF1 attenuated the inhibitory effects of FAM3A on gluconeogenesis and lipid accumulation in hepatocytes. Akt targets FOXO1 to inhibit the transcriptions of key gluconeogenic and lipogenic genes [66, 67]. PANX1 overexpression activated Akt to promote FOXO1 nuclear exclusion, and this effect was blocked by PBN and suramin. In addition, PBN treatment also abrogated the roles of FAM3A in suppressing gluconeogenesis and ameliorating lipid deposition. Overexpression of FAM3A in livers could not rescue the glucolipid metabolic disorder observed in PANX1-deficient mice. Collectively, the effects of FAM3A on promoting ATP release to suppress gluconeogenesis and lipogenesis were mediated by PANX1. Moreover, it should be noted that the disturbed cross-regulation loop between HSF1 and FAM3A pathways might play vital roles in the development of metabolic disorders. Under obese conditions, the stimulatory effects of FFAs on PANX1 expression prevail over the inhibition effect caused by reduced levels of FAM3A in the liver, which is likely an important compensatory mechanism against the decrease in ATP synthesis.

In the past decade, we and others have established the critical roles of the mitochondrial protein FAM3A in regulating various important biological processes in the liver, pancreatic β cells, vascular smooth muscle cells, endothelial cells, neural cells, and other cells by stimulating ATP synthesis and release [16–23, 25, 68, 69]. Recently, we identified FAM3A as a novel component of the ATPS complex, and it directly activates ATPS activity to enhance ATP synthesis and suppress reactive oxygen species (ROS) production. In this investigation, we elucidated the mechanisms underlying FAM3A-induced ATP release in hepatocytes. Together with our previous findings, the discoveries from this current study have shed light on unveiling the panorama of ATP synthesis and release in controlling hepatic glucolipid metabolism. Children and adults with diabetes exhibited decreased blood levels of ATP [70, 71]. Meanwhile, arterial and venous plasma ATP levels in individuals with type 2 diabetes significantly reduced during exercise [71]. These findings suggested that impaired ATP release played a crucial role in the development of metabolic disorders. We had previously demonstrated that FAM3A-induced ATP release activates UCP1 expression and thermogenesis in brown adipose tissue (BAT) [22]. Therefore, we speculate that PANX1-mediated ATP release from the liver may also act on other tissues such as BAT, which could at least partially explain changes in body weight observed upon PANX1 overexpression or inhibition in mice. Given the crucial roles played by repressed ATP synthesis and release in the pathogenesis of diabetes and NAFLD, our findings reveal that targeting FAM3A represents an attractive strategy for combating metabolic disorders.

## Conclusions

In conclusion, PANX1-mediated ATP release is critical for inhibiting hepatic gluconeogenesis and lipogenesis. Under obese conditions, the upregulation of hepatic PANX1 expression via MafK repression serves as an important compensatory mechanism for maintaining glucose and lipid homeostasis. FAM3A activates HSF1 to induce PANX1 expression, thereby facilitating FAM3A-induced ATP release and its subsequent regulatory effects on glucolipid metabolism in hepatocytes (Fig. 9i). Overall, activation of the insulin-independent FAM3A-PANX1-ATP-P2Y receptors signaling pathway is effective in treating metabolic disorders.

### Supplementary Information

**Additional file 1: Fig. S1** Overexpression of pannexin 1 (PANX1) inhibited glucose production and lipid accumulation in HepG2 cells. **Fig. S2** Verifying the efficiency of AAV-shPANX1 in mouse hepatocytes. **Fig. S3** Hepatic PANX1 inhibition aggravated HFD-induced dysregulated glucolipid metabolism in HFD-fed mice. **Fig. S4** PANX1-deficient mice exhibited impaired glucose tolerance fed on a normal diet. **Fig. S5** Treatment with PBN or suramin blunted PANX1-mediated regulatory effects on glucolipid metabolism in HepG2 cells. **Fig. S6** Free fatty acids upregulated PANX1 expression by inhibiting MafK in HepG2 cells. **Fig. S7** FAM3A overexpression induced PANX1 expression in HepG2 cells. **Fig. S8** HSF1 activated the expression of PANX1 in HepG2 cells. **Fig. S9** PBN treatment blocked FAM3A-promoted ATP release in HepG2 cells. **Fig. S10** Liver FAM3A overexpression failed to improve the impaired glucose tolerance in PANX1-deficient mice. **Table S1** Clinical parameters of individuals with or without NAFLD. **Table S2** siRNA sequence against mouse MafK mRNAs. **Table S3** List of oligonucleotide primer pairs used in Real-time PCR analysis. **Table S4** MS data of CaM co-immunoprecipitation. **Table S5** MS data of DNA pull-down.

**Additional file 2:** RNA-sequencing data.

## Data Availability

All data are available in the main text or the Supplementary materials.

## Abbreviations

AAV: Adeno-associated virus
Akt: Protein kinase B
AP1: Activator protein-1
ATP: Adenosine triphosphate
ATPS: ATP synthase
CaM: Calmodulin
Co-IP: Co-immunoprecipitation
CX: Connexin
FAM3: Family with sequence similarity 3
FAM3A: Family with sequence similarity 3 member A
FAS: Fatty acid synthase
FFAs: Free fatty acids
FOXO1: Forkhead box protein O1
G-6-Pase: Glucose-6-phosphatase
HFD: High-fat diet
ITT: Insulin tolerance test
JNK: C-Jun N-terminal kinase
MafK: V-maf musculoaponeurotic fibrosarcoma oncogene homolog K
MRI: Magnetic resonance imaging
MS: Mass spectrometry
NAFLD: Non-alcoholic fatty liver disease
ND: Normal diet
OGTT: Oral glucose tolerance test
PANX: Pannexin
PANX1: Pannexin 1
PBN: Probenecid
PEPCK: Phosphoenolpyruvate carboxykinase
PRs: Purinergic receptors
PTT: Pyruvate tolerance test
TG: Triglyceride
