# Supplementary material for: PANX1-mediated ATP release confers FAM3A’s suppression effects on hepatic gluconeogenesis and lipogenesis
Source: Mil Med Res. 2024 Jun 27;11:41. doi: 10.1186/s40779-024-00543-6 (PMC11210080; doi:10.1186/s40779-024-00543-6)
Supplement: Supplementary file 1 — Additional file 1: Fig. S1 Overexpression of pannexin 1 (PANX1) inhibited glucose production and lipid accumulation in HepG2 cells. Fig. S2 Verifying the efficiency of AAV-shPANX1 in mouse hepatocytes. Fig. S3 Hepatic PANX1 inhibition aggravated HFD-induced dysregulated glucolipid metabolism in HFD-fed mice. Fig. S4 PANX1-deficient mice exhibited impaired glucose tolerance fed on a normal diet. Fig. S5 Treatment with PBN or suramin blunted PANX1-mediated regulatory effects on glucolipid metabolism in HepG2 cells. Fig. S6 Free fatty acids upregulated PANX1 expression by inhibiting MafK in HepG2 cells. Fig. S7 FAM3A overexpression induced PANX1 expression in HepG2 cells. Fig. S8 HSF1 activated the expression of PANX1 in HepG2 cells. Fig. S9 PBN treatment blocked FAM3A-promoted ATP release in HepG2 cells. Fig. S10 Liver FAM3A overexpression failed to improve the impaired glucose tolerance in PANX1-deficient mice. Table S1 Clinical parameters of individuals with or without NAFLD. Table S2 siRNA sequence against mouse MafK mRNAs. Table S3 List of oligonucleotide primer pairs used in Real-time PCR analysis. Table S4 MS data of CaM co-immunoprecipitation. Table S5 MS data of DNA pull-down. [file 40779_2024_543_MOESM1_ESM.pdf]

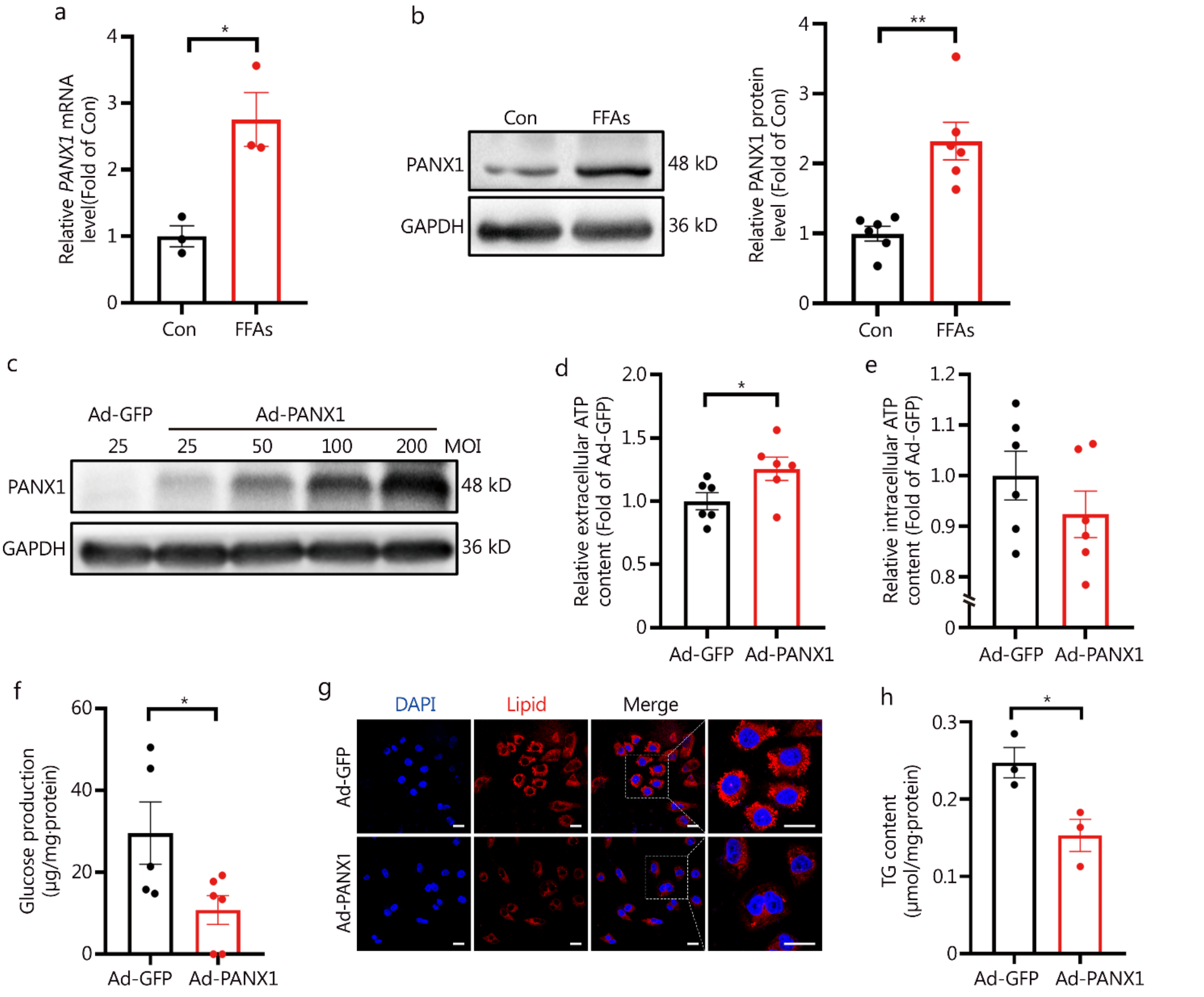

Fig. S1 Overexpression of pannexin 1 (PANX1) inhibited glucose production and lipid accumulation in HepG2 cells. **a** Treatment with FFAs increased the *PANX1* mRNA level in HepG2 cells (n = 3). **b** Treatment with FFAs increased PANX1 protein level in HepG2 cells. The left panel showed representative gel images of protein levels, and the right panel presented quantitative data (n = 6). **c** Verification of the efficiency of PANX1-overexpressing adenovirus in HepG2 cells. The effects of PANX1 overexpression on extracellular (**d**) and intracellular (**e**) ATP content in HepG2 cells (n = 6). **f** Overexpression of PANX1 inhibited glucose production in HepG2 cells, n = 5 (Ad-GFP) and 6 (Ad-PANX1). **g** Representative confocal lipid staining images of Ad-GFP- or Ad-PANX1-infected HepG2 cells. Scale bar = 25 µm. **h** PANX1 overexpression decreased TG content in FFAs-treated HepG2 cells (n = 3). $^{*}P < 0.05$ or $^{**}P < 0.01$. Ad-GFP Ad-GFP-infected HepG2 cells, Ad-PANX1 Ad-PANX1-infected HepG2 cells, MOI multiplicity of infection, Con control, ATP adenosine triphosphate, TG triglyceride

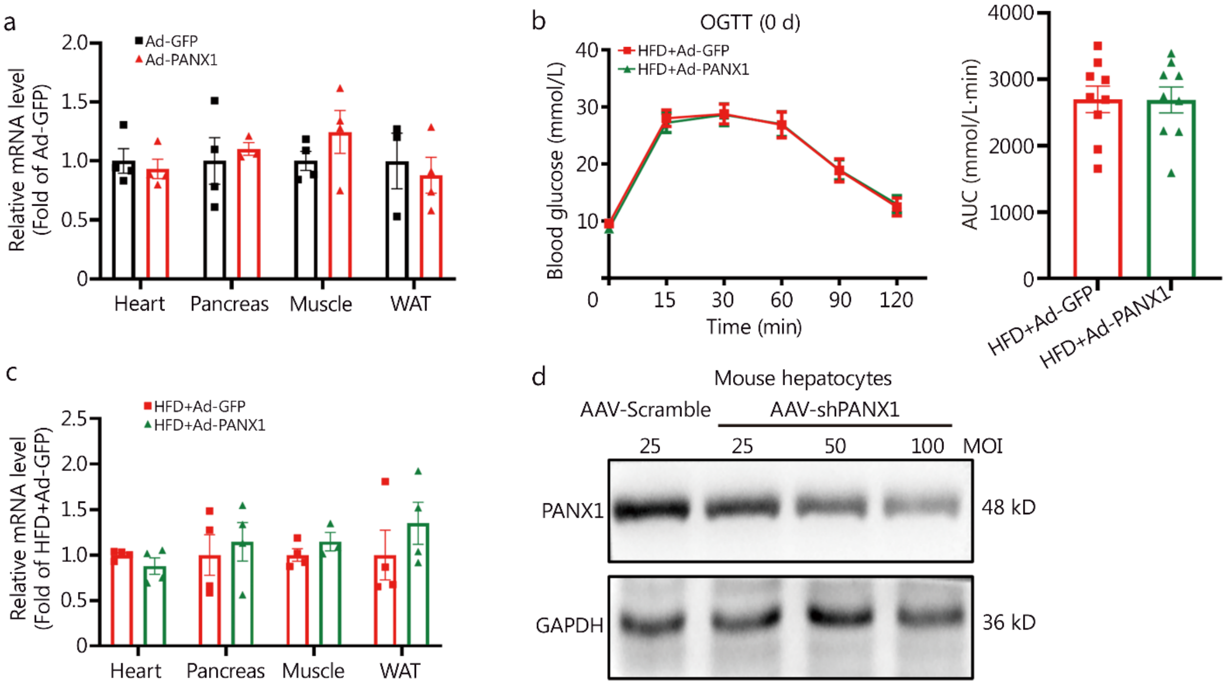

Fig. S2 Verifying the efficiency of AAV-shPANX1 in mouse hepatocytes. **a** Relative pannexin 1 (*PANX1*) mRNA levels in main metabolic tissues *db/db* mice injected with Ad-PANX1 or Ad-GFP, from left to right, *n* = 4, 4, 4, 3, 4, 4, 3 and 4, respectively. **b** Oral glucose tolerance test (OGTT) had no significant difference between two sets of mice before adenoviral injection in high-fat diet (HFD)-fed mice. The left panel showed OGTT curves and the right panel presented the area under the curve (AUC) data (*n* = 9). **c** Relative *PANX1* mRNA levels in main metabolic tissues of HFD injected with Ad-PANX1 or Ad-GFP, *n* = 4 (HFD + Ad-GFP), from left to right, *n* = 4, 4, 3 and 4 (HFD + Ad-PANX1), respectively. **d** Verification of the efficiency of AAV-shPANX1 in mouse hepatocytes. WAT white adipose tissues, Ad-GFP Ad-GFP-infected HepG2 cells, Ad-PANX1 Ad-PANX1-infected HepG2 cells, AAV-Scramble AAV-Scramble-infected mouse hepatocytes, AAV-shPANX1 AAV-shPANX1-infected mouse hepatocytes

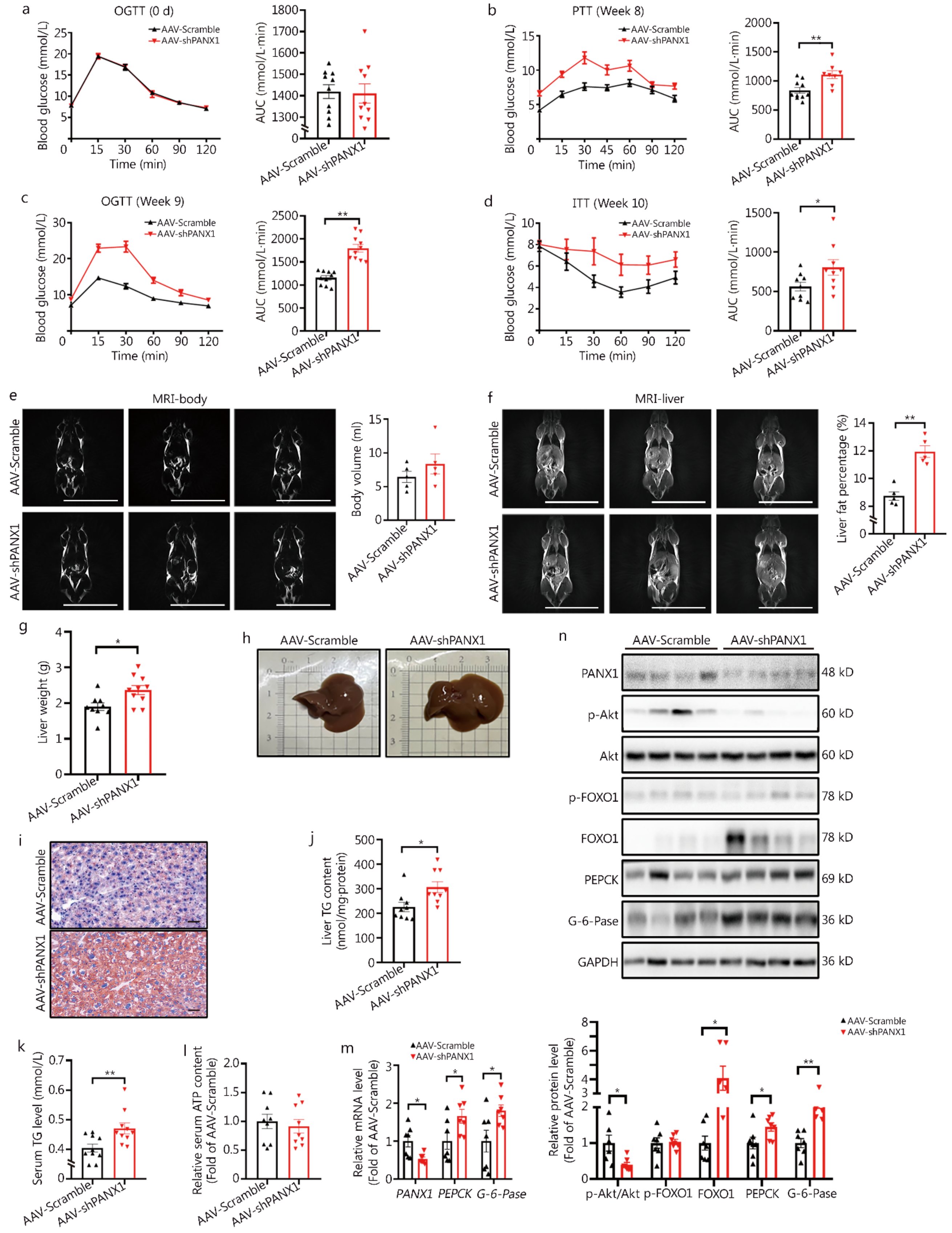

Fig. S3 Hepatic PANX1 inhibition aggravated HFD-induced dysregulated glucolipid metabolism in HFD-fed mice. **a** Oral glucose tolerance test (OGTT) showed no difference between the two sets of mice prior to adeno-associated viral (AAV) injection in high-fat diet (HFD)-fed mice (n = 10). The left panel showed OGTT curves and the right

panel presented the area under the curve (AUC) data. Mice were transduced with AAV-GFP or AAV-shPANX1 and then subjected to HFD feeding. **b** The left panel showed pyruvate tolerance test (PTT) curves on week 8 post-AAV injection in HFD-fed mice, and the right panel presented AUC data, n = 10 (AAV-Scramble) and 8 (AAV-shPANX1). **c** The left panel showed OGTT curves on week 9 post-AAV injection in HFD-fed mice, and the right panel presented AUC data (n = 10). **d** The left panel showed insulin tolerance test (ITT) curves on week 10 post-AAV injection in HFD-fed mice, and the right panel displayed AUC data (n = 9). **e** The left panel presented representative magnetic resonance imaging (MRI) images of whole-body fat of HFD-fed mice injected with AAV-Scramble or AAV-shPANX1 at 11 weeks, and the right panel displayed quantitative data. Scale bar = 5 cm (n = 5). **f** The left panel presented representative MRI images of liver fat of HFD-fed mice injected with AAV-Scramble or AAV-shPANX1 at 11 weeks, and the right panel showed quantitative data (n = 5). Scale bar = 5 cm. Liver weight (**g,** from left to right, n = 9 and 10) of mice on week 13 (sacrifice) post-viral-injection. Morphological observation (**h**) and oil red O staining analyses (**i**) of AAV-Scramble- or AAV-shPANX1-injected mouse livers (n = 5). Scale bar = 50 μm. Hepatic (**j,** n = 9) and serum (**k,** n = 10) triglyceride (TG) levels of AAV-Scramble- or AAV-shPANX1-injected mice. **l** Serum adenosine triphosphate (ATP) content in the two groups of mice (n = 9). **m** Relative mRNA levels of gluconeogenic genes in the two sets of mouse livers (n = 7). **n** The upper panel showed representative gel images of gluconeogenic proteins in mouse livers, and the lower panel displayed quantitative data, from left to right, n = 6, 7, 7, 7, 7, 6, 7, 7, 7 and 7, respectively. $^{*}P < 0.05$ or $^{**}P < 0.01$. AAV-Scramble AAV-Scramble-injected HFD-fed mice, AAV-shPANX1 AAV-shPANX1-injected HFD-fed mice, Akt protein kinase B, FOXO1 forkhead box protein O1, PEPCK phosphoenolpyruvate carboxykinase, G-6-Pase glucose-6-phosphatase

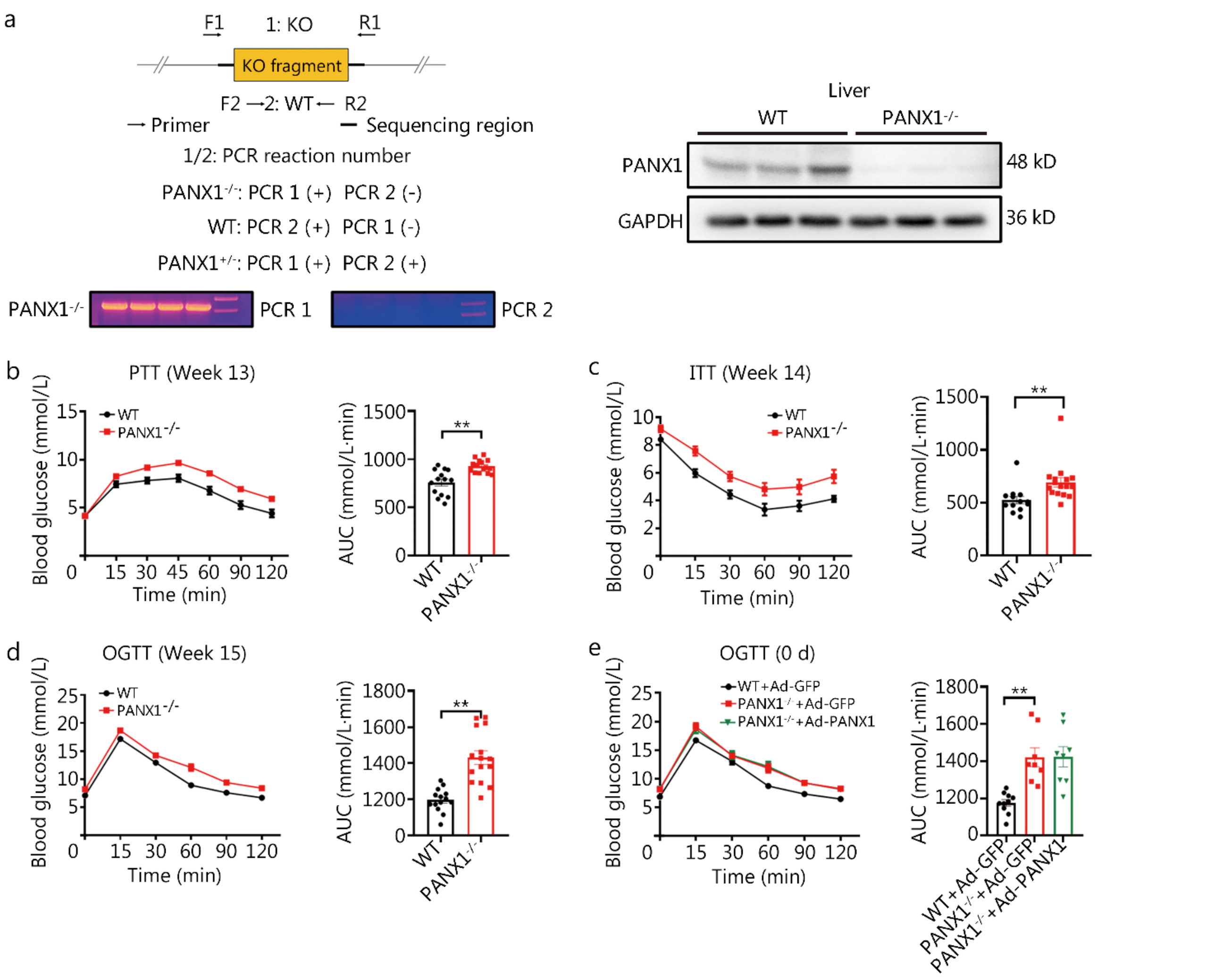

Fig. S4 PANX1-deficient mice exhibited impaired glucose tolerance fed on a normal diet. **a** The generation (*n* = 4) and characterization (*n* = 3) of Pannexin 1 (*PANX1*) knockout mice. **b** The left panel displayed pyruvate tolerance test (PTT) curves of wild type (WT) and PANX1-deficient mice at 13-week old, and the right panel showed area under the curve (AUC) data, *n* = 14 (WT) and 15 ($PANX1^{-/-}$). **c** The left panel displayed insulin tolerance test (ITT) curves of WT and PANX1-deficient mice at 14-week old, and the right panel presented AUC data *n* = 13 (WT) and 16 ($PANX1^{-/-}$). **d** The left panel presented oral glucose tolerance test (OGTT) curves of WT and PANX1-deficient mice at 15-week old, and the right panel showed AUC data, *n* = 14 (WT) and 15 ($PANX1^{-/-}$). **e** The left panel displayed OGTT curves of WT and PANX1-deficient mice at 16-week old before adenoviral injection (Day 0), and the right panel showed AUC data, *n* = 10 (WT + Ad-GFP), 8 ($PANX1^{-/-}$ + Ad-GFP) and 8 ($PANX1^{-/-}$ + Ad-PANX1). $^{**}P < 0.01$. KO knock out, PCR polymerase chain reaction, WT wild-type, $PANX^{-/-}$ PANX1-deficient mice, WT + Ad-GFP Ad-GFP-injected wild type mice, $PANX^{-/-}$ + Ad-GFP Ad-GFP-injected PANX1-deficient mice, $PANX^{-/-}$ + Ad-PANX1 Ad-PANX1-injected PANX1-deficient mice

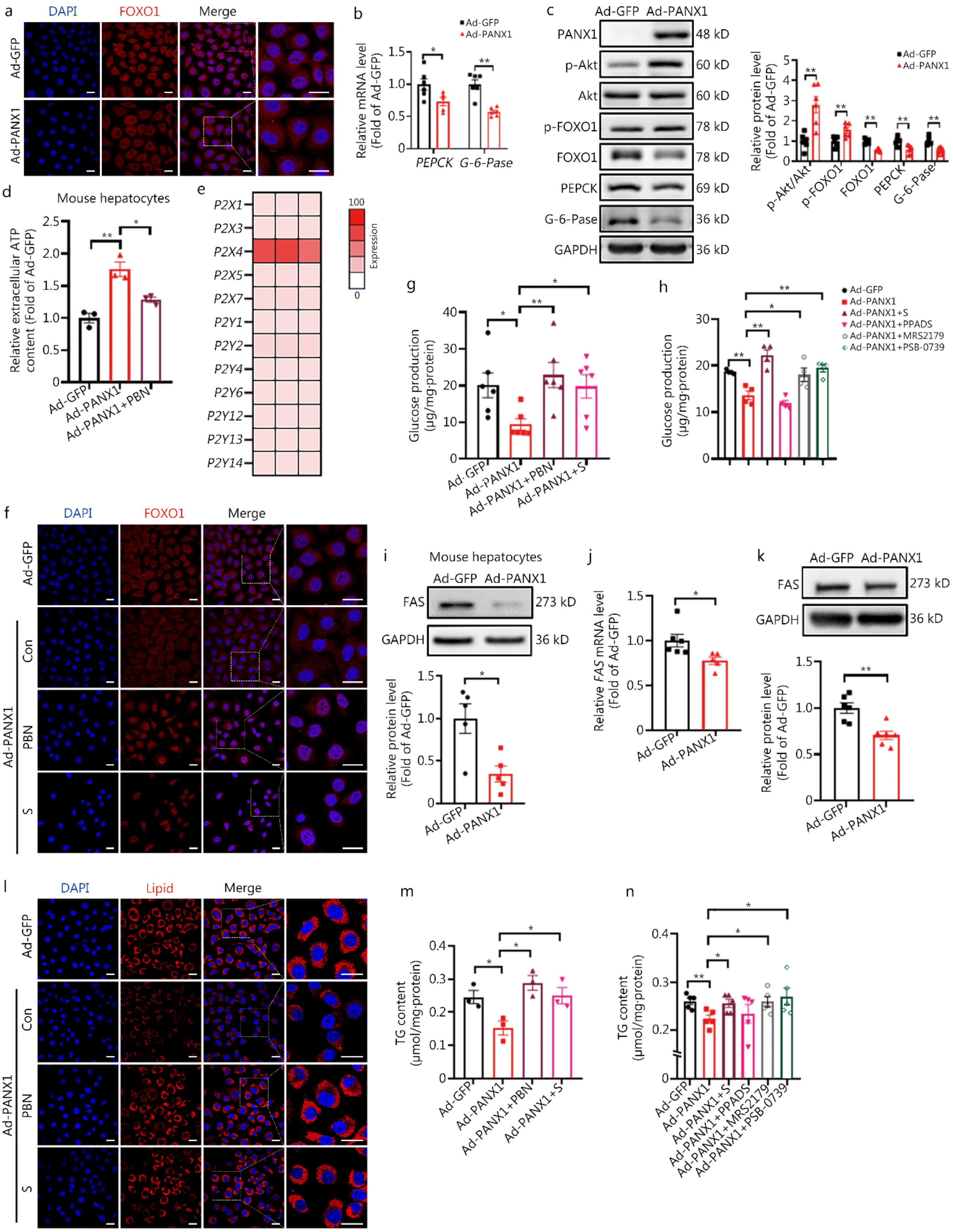

Fig. S5 Treatment with PBN or suramin blunted PANX1-mediated regulatory effects on glucolipid metabolism in HepG2 cells. **a** Representative immunofluorescent staining images of forkhead box protein O1 (FOXO1) in Ad-GFP- or Ad-PANX1-infected HepG2 cells (n = 3). Scale bar =25 μm. **b** Relative mRNA levels of gluconeogenic genes in

Ad-GFP- or Ad-PANX1-infected HepG2 cells, from left to right, $n = 6$, 5, 6, and 6, respectively. **c** The left panel showed representative gel images of gluconeogenic proteins in Ad-GFP- or Ad-PANX1-infected HepG2 cells, and the right panel displayed quantitative data ($n = 6$). **d** PBN treatment reversed PANX1-induced increase in extracellular adenosine triphosphate (ATP) content in mouse hepatocytes ($n = 3$). **e** Determining the level profile of P2 receptors using RNA-sequencing in primary hepatocytes. The panel showed the heat map based on transcripts per million (TPM) ($n = 3$). Treatment with PANX1 inhibitor PBN or P2 receptor inhibitor suramin blunted PANX1-induced FOXO1 nuclear exclusion of FOXO1 (**f,** $n = 3$) and inhibition of glucose production (**g,** $n = 6$) in HepG2 cells. Scale bar = 25 μm. **h** The effect of PANX1 inhibitor PBN, pan P2 receptor inhibitor suramin, pan P2X inhibitor PPADS, specific P2Y1 inhibitor MRS2179 or specific P2Y12 inhibitor PSB-0739 on the glucose production in mouse hepatocytes ($n = 4$). **i** PANX1 overexpression inhibited FAS protein level in mouse hepatocytes. The upper panel presented representative gel images, and the lower panel showed quantitative data ($n = 5$). **j** PANX1 overexpression inhibited *FAS* mRNA level in HepG2 cells, $n = 6$ (Ad-GFP) and 5 (Ad-PANX1). **k** PANX1 overexpression decreased FAS protein level in HepG2 cells. The upper panel displayed representative gel images, and the lower panel presented quantitative data ($n = 6$). Treatment with PBN or suramin reversed PANX1-induced decrease in lipid accumulation (**l**) and triglyceride (TG) levels (**m**) in mouse hepatocytes ($n = 3$). Scale bar = 25 μm. **n** The effect of PANX1 inhibitor PBN, pan P2 receptor inhibitor suramin, pan P2X inhibitor PPADS, specific P2Y1 inhibitor MRS2179 or specific P2Y12 inhibitor PSB-0739 on lipid deposition in mouse hepatocytes ($n = 5$). $^{*}P < 0.05$ or $^{**}P < 0.01$. Con control, Ad-GFP Ad-GFP-infected HepG2 cells, Ad-PANX1 Ad-PANX1-infected HepG2 cells, Akt protein kinase B, FOXO1 forkhead box protein O1, PEPCK phosphoenolpyruvate carboxykinase, G-6-Pase glucose-6-phosphatase, PBN pannexin 1 (PANX1) inhibitor probenecid, S suramin

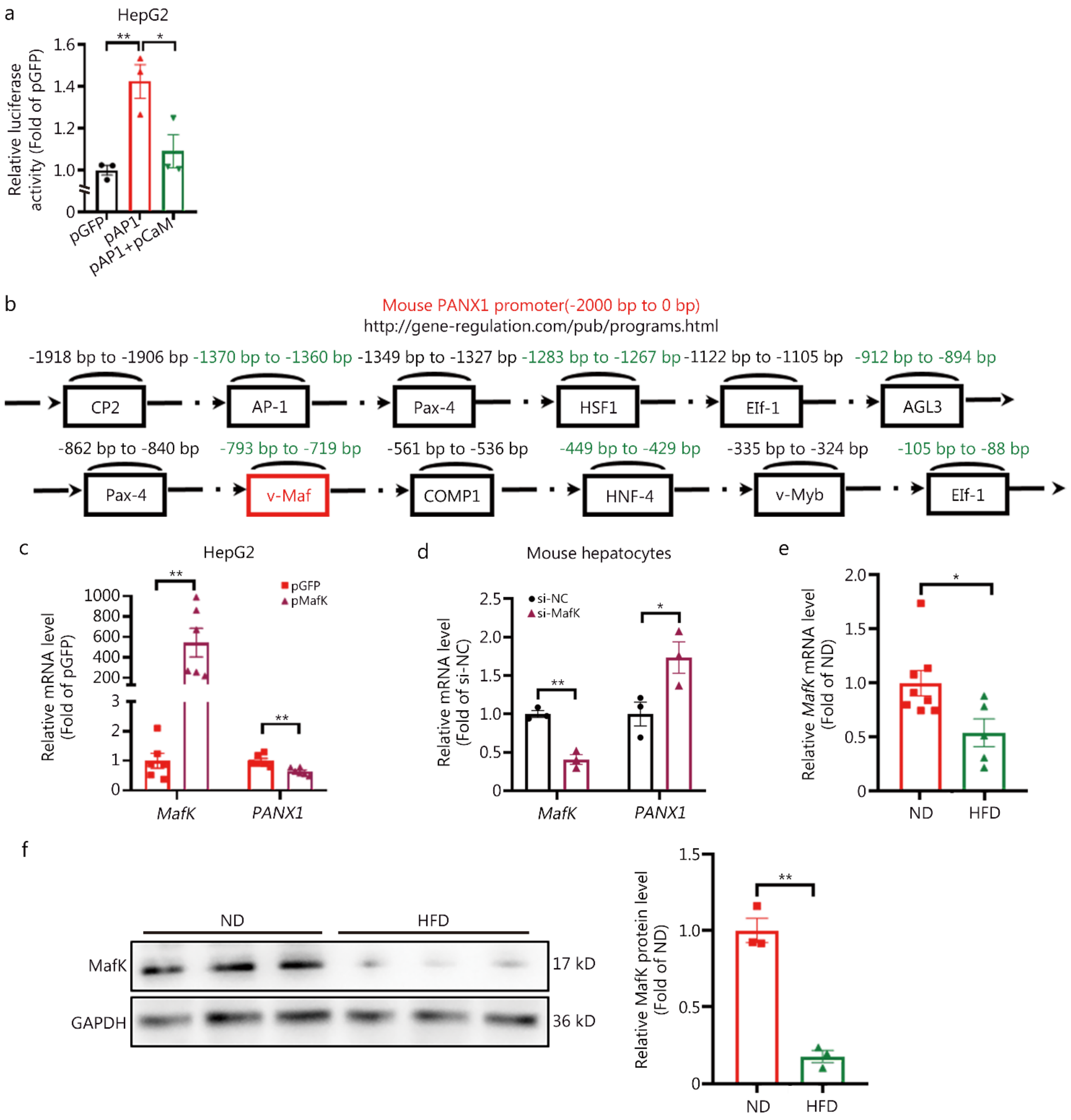

Fig. S6 Free fatty acids upregulated PANX1 expression by inhibiting MafK in HepG2 cells. **a** Calmodulin (CaM) overexpression reversed activator protein-1 (AP1)-promoted activation of the fatty acid synthase (*FAS*) gene promoter in HepG2 cells (n = 3). **b** The potential binding sites for the transcription factors in mouse pannexin 1 (*PANX1*) gene promoters were analyzed in http://www.gene-regulation.com/pub/databases.html/. The potential binding sites are highly specific for certain transcription factors with high prediction scores presented. **c** Plasmid-mediated v-maf musculoaponeurotic fibrosarcoma oncogene homolog K (MafK) overexpression inhibited the mRNA level of *PANX1* in HepG2 cells (n = 6). **d** siRNA silencing of *MafK* enhanced *PANX1* mRNA level in mouse hepatocytes (n = 3). **e** The *MafK* mRNA level was decreased in high-fat diet (HFD) mouse livers, n = 8 (ND) and 5 (HFD). **f** The MafK protein level was reduced in HFD mouse livers. The left panel showed representative gel images and the right panel

displayed quantitative data ($n = 3$). $^{*}P < 0.05$, $^{**}P < 0.01$. CP2 alpha-globin transcription factor CP2, Pax-4 paired box 4, HSF1 heat shock factor 1, Elf-1 ETS-related transcription factor Elf-1, AGL3 agamous-like MADS-box protein AGL3, v-Maf v-maf avian musculoaponeurotic fibrosarcoma oncogene homolog, COMP1 cooperates with myogenic proteins 1, HNF-4 hepatocyte nuclear factor-4, v-Myb v-myb avian myeloblastosis viral oncogene homolog

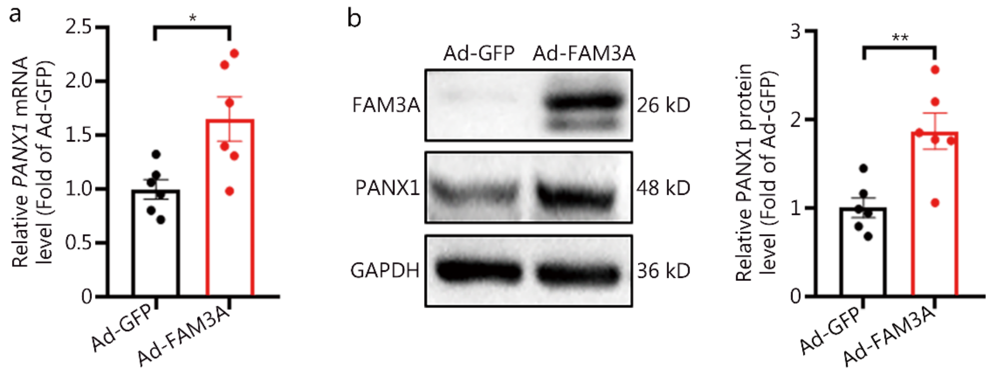

Fig. S7 FAM3A overexpression induced PANX1 expression in HepG2 cells. **a** Family with sequence similarity 3 member A (FAM3A) overexpression increased pannexin 1 (*PANX1*) mRNA level in HepG2 cells. **b** Overexpression of FAM3A upregulated PANX1 protein level in HepG2 cells. The left panel showed representative gel images and the right panel presented quantitative data. $n = 6$ for the corresponding experiments. $^{*}P < 0.05$, $^{**}P < 0.01$. Ad-GFP Ad-GFP-infected HepG2 cells, Ad-FAM3A Ad-FAM3A-infected HepG2 cells

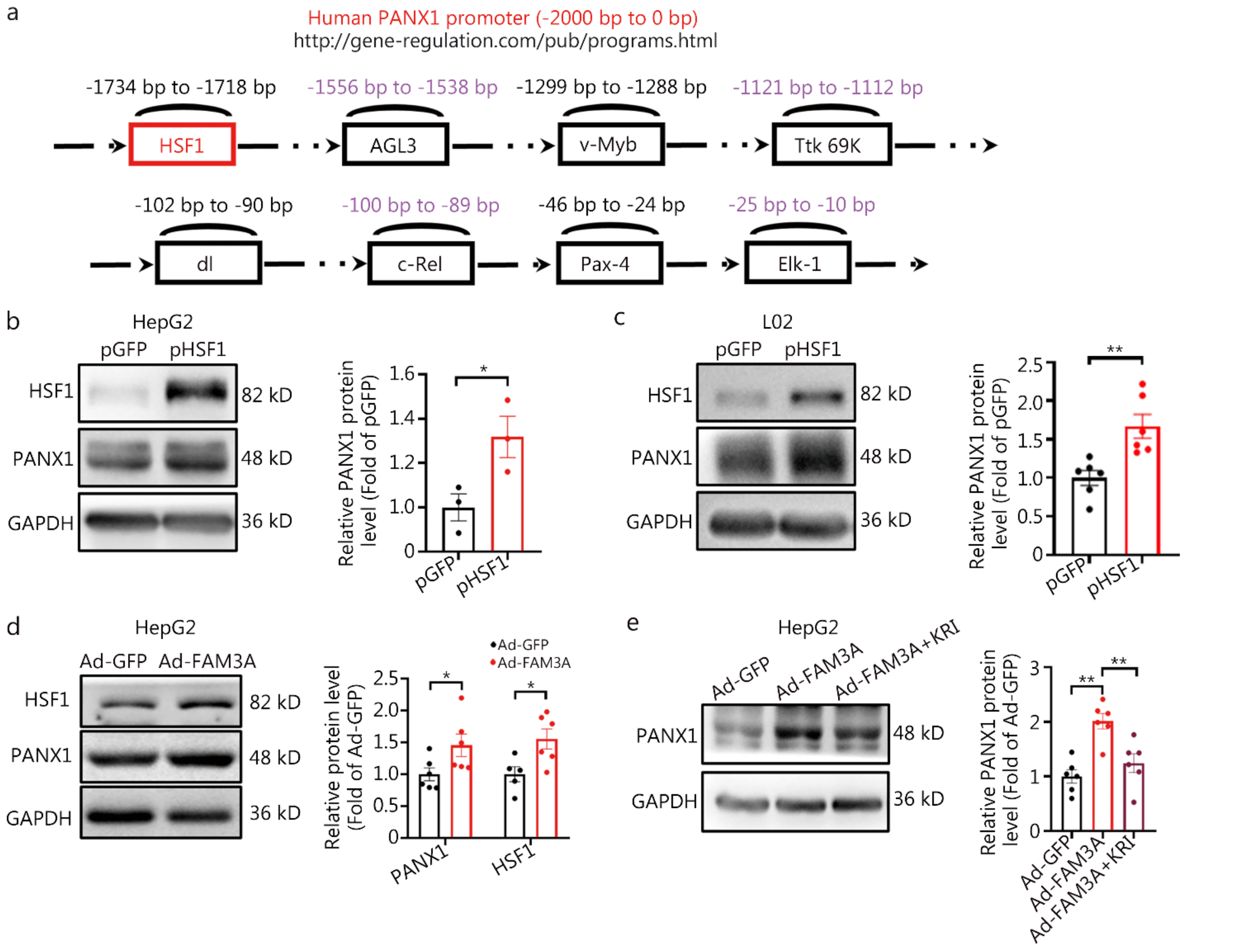

Fig. S8 HSF1 activated the expression of PANX1 in HepG2 cells. **a** The binding sites for transcription factors in human pannexin 1 (*PANX1*) gene promoters were analyzed at http://www.gene-regulation.com/pub/databases.html/. The potential binding sites are highly specific for certain transcription factors with high prediction scores presented. Plasmid overexpression of heat shock factor 1 (HSF1) increased PANX1 protein levels in HepG2 (**b,** n = 3) and L02 (**c,** n = 6) cells. The left panel showed representative gel images and the right panel presented quantitative data. **d** FAM3A overexpression enhanced HSF1 and PANX1 protein levels in HepG2 cells. The left panel presented representative gel images, and the right panel displayed quantitative data, from left to right, n = 6, 6, 5, and 6, respectively. **e** Treatment with HSF1 inhibitor blocked FAM3A-triggered PANX1 protein level in HepG2 cells (n = 6). The left panel presented representative gel images, and the right panel displayed quantitative data. $^{*}P < 0.05$, $^{**}P < 0.01$. AGL3 agamous-like MADS-box protein AGL3, v-Myb v-myb avian myeloblastosis viral oncogene homolog, Ttk 69K tramtrack 69K, dl embryonic polarity protein dorsal, c-Rel proto-oncogene c-Rel, Pax-4 paired box 4, Elk-1 ETS domain-containing protein Elk-1, KRI KRIBB11

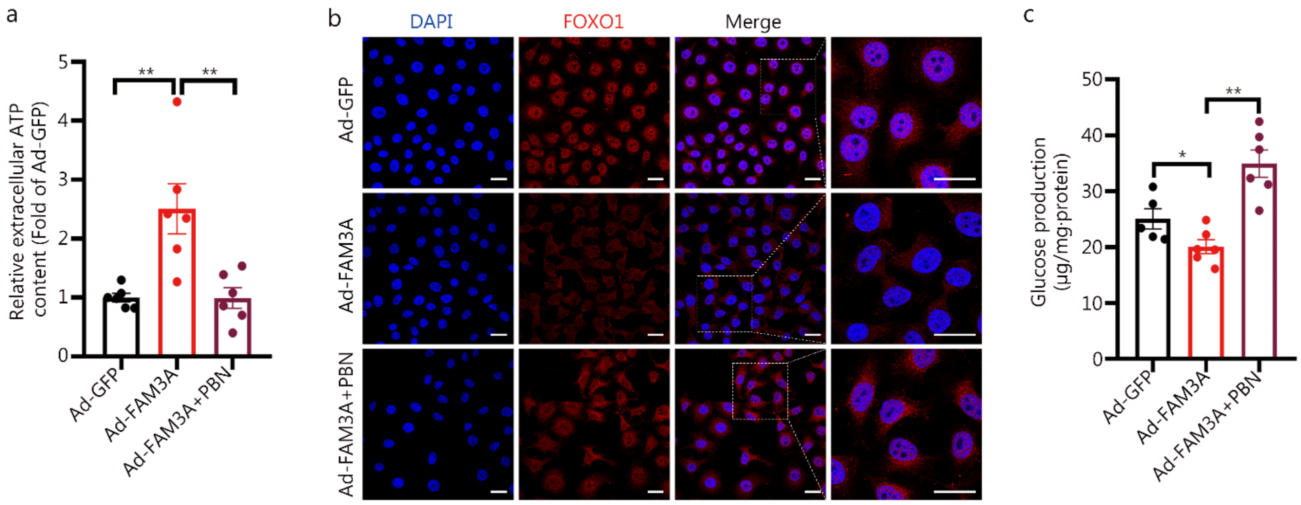

Fig. S9 PBN treatment blocked FAM3A-promoted ATP release in HepG2 cells. **a** PBN treatment inhibited FAM3A-promoted ATP release in HepG2 cells (n = 6). PBN treatment blocked FAM3A-induced forkhead box protein O1 (FOXO1) nuclear exclusion (**b,** n = 3) and inhibition of glucose production (**c,** from left to right, n = 5, 6, and 6) in HepG2 cells. Scale bar = 25 μm. $^{*}P < 0.05$, $^{**}P < 0.01$. PBN pannexin 1 (PANX1) inhibitor probenecid, ATP adenosine triphosphate, Ad-GFP Ad-GFP-infected HepG2 cells, Ad-FAM3A Ad-FAM3A-infected HepG2 cells

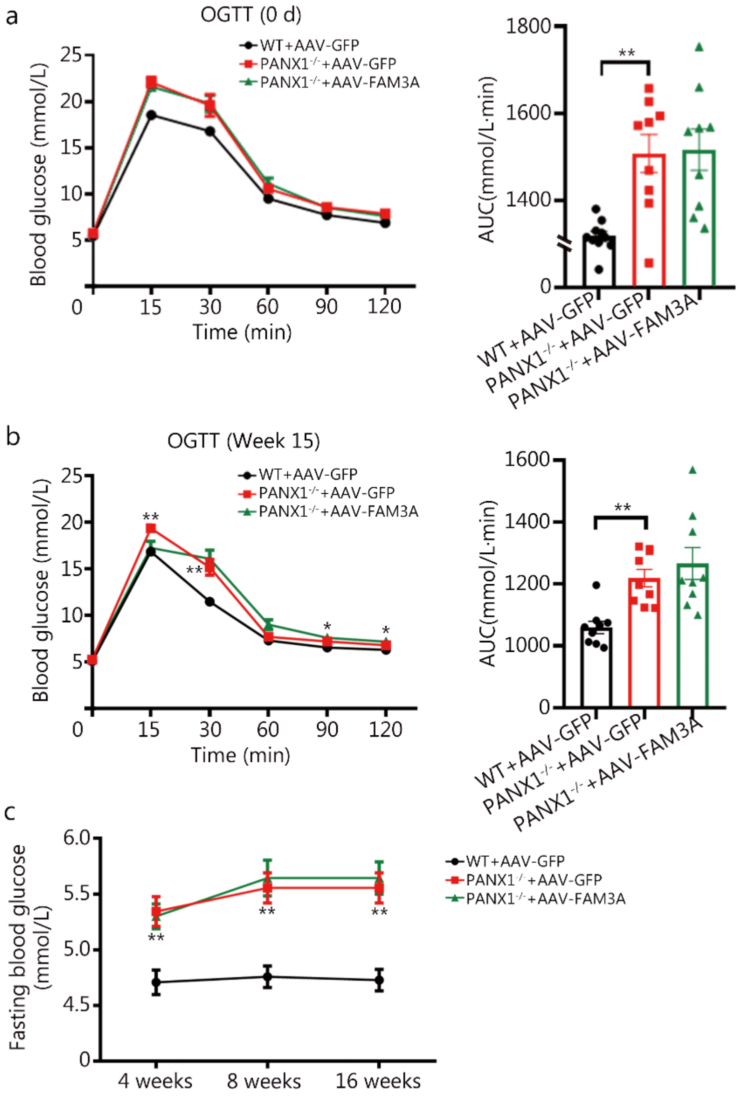

Fig. S10 Liver FAM3A overexpression failed to improve the impaired glucose tolerance in PANX1-deficient mice. **a** Oral glucose tolerance test (OGTT) exhibited no difference between the two sets of PANX1-deficient mice prior to AAV injection. The left panel displayed OGTT curves, while the right panel presented AUC data, n = 10 (WT + AAV-GFP), 9 (PANX1$^{-/-}$ + AAV-GFP) and 9 (PANX1$^{-/-}$ + AAV-FAM3A). **b** The left panel showed OGTT curves on week 15 post-AAV injection in PANX1-deficient mice, and the right panel displayed area under the curve (AUC) data (n = 9). **c** Fasting blood glucose on week 4, 8 and 16 post AAV injection in PANX1-deficient mice, n = 10 (WT + AAV-GFP), 9 (PANX1$^{-/-}$ + AAV-GFP) and 9 (PANX1$^{-/-}$ + AAV-FAM3A). In panels b and c, the * and ** indicate the comparison between WT + AAV-GFP and PANX1$^{-/-}$ + AAV-GFP. $^{*}P < 0.05$, $^{**}P < 0.01$. WT wild-type, PANX$^{-/-}$ PANX1-deficient mice, FAM3A family with sequence similarity 3 member A, PANX$^{-/-}$ + AAV-GFP AAV-GFP-injected PANX1-deficient mice, PANX$^{-/-}$ + AAV-FAM3A AAV-FAM3A-injected PANX1-deficient mice

Table S1 Clinical parameters of individuals with or without non-alcoholic fatty liver disease (NAFLD)

Group	Age (year)	Gender	Height (cm)	Weight (kg)	ALT (U/L)	AST (U/L)	Albumin (g/L)	Total cholesterol (mmol/L)	Triglyceride (mmol/L)	HDL-C (mmol/L)	LDL-C (mmol/L)	Degree of steatosis
	65	Female	155	62	11	23	45.5	5.16	0.70	2.10	2.72	FO
Control	58	Female	155	80	21	21	43.6	3.68	1.19	1.36	1.75	FO
	72	Male	179	80	15	18	41.6	5.54	1.44	1.36	3.31	FO
	57	Female	160	72	65	60	39.1	5.33	1.41	1.12	3.25	F1-F2
NAFLD	60	Male	181	90	16	19	43.8	4.7	0.93	1.15	2.78	F1-F2
	54	Female	159	54	11	21	43.8	6	2.28	1.07	4.11	F1-F2

ALT alanine aminotransferase, *AST* aspartate aminotransferase, *HDL-C* high-density lipoprotein cholesterol, *LDL-C* low-density lipoprotein cholesterol

Table S2 siRNA sequence against mouse *MafK* mRNAs

**Name**	**Sense/ antisense**	**Sequences**
si-MafK (Mus musculus)	Sense 1	5'-UCUUAGCGAUGAUGAGCUGGU(dT)(dT)-3'
	Anti-sense 1	5'-ACCAGCUCAUCAUCGCUAAGA(dT)(dT)-3'
	Sense 2	5'-CCACCACCAGUGUCAUCACCA(dT)(dT)-3'
	Anti-sense 2	5'-UGGUGAUGACACUGGUGGUGG(dT)(dT)-3'
	Sense 3	5'-CGCUCCAAGUAUGAGGCCCUA(dT)(dT)-3'
	Anti-sense 3	5'-UAGGGCCUCAUACUUGGAGCG(dT)(dT)-3'

Table S3 List of oligonucleotide primer pairs used in Real-time PCR analysis

Gene	Forward primer (5'-3')	Reverse primer (5'-3')
PANX1 (H)	CCTGAGAAACGACAGCACC	TGTAGACAACCACGGGAGC
PANX1 (M)	AGCATCAAATCAGGCGTCC	TGTAGACGACCACGGGAATC
PANX2 (H)	CCCAGAGCCAGGGAAGAG	GGCGACAAGGAGAAAGTGC
PANX2 (M)	CATACCCGCCACTTCTCC	CCCAGCCCACATTCCTC
PANX3 (H)	TCATCATCAGCGAACTGG	CGTTCTTTCCGAGCCTT
PANX3 (M)	GGAACTGCCCCTGGATA	CGGAGACCCTGATGAGAA
CX26 (M)	ATCTGGCTCACGGTCCTCTT	GGAAGTGGTGGTCGTAGCATA
CX32 (M)	GTGGACCTATGTCATCAGT	GGAAGGCTTCACACTTGACC
CX43 (M)	GGTGATGAACAGTCTGCCTT	GTGAGCCAAGTACAGGAGTGT
FAM3A (H)	GTGTCACATGGATCGTGGTC	TGCTCAATCAGCATCTTGTCC
FAM3A (M)	TCATGAGCAGCGTCAAAGA	AGGGTACCTTCATGCAGTGG
PEPCK (H)	ATGGCCGCATTGTACCGCC	TCACATTTTGTGCACACGTCTC
PEPCK (M)	ATCTTTGGTGGCCGTAGACCT	CCGAAGTTGTAGCCGAAGAA
G-6-Pase (H)	GGCATTGCTGTTGCAGAAACT	AGGTCTACACCCAGTCCCTTGA
G-6-Pase (M)	AGGAAGGATGGAGGAAGGAA	TGGAACCAGATGGGAAAGAG
FAS (H)	GGAGAACCAGACCCCAGAGT	CACAGAGGAGAAGACCACAAA
FAS (M)	CTGCCACAACTCTGAGGACA	CGGATCACCTTCTTGAGAGC
SREBP1 (M)	ACTTCTGGAGACATCGCAAAC	GGTAGACAACAGCCGCATC
ACC (M)	TGGTCGTGACTGCTCTGTGC	GTAGCCGAGGGTTCAGTTCC
SCD1 (M)	ATGTGCCAGAGGAGCTGAGT	TGATCCACTGTTGCTTCTGC
PPARγ (M)	ACCACTCGCATTCCTTT	CACAGACTCGGCACTCA
CHREBP (M)	TTACTGGAAGCGGCGCATCG	CCAAGCAGCACAGGCACCAC
LXR (M)	TGCCATCAGCATCTTCTCTG	GGCTCACCAGCTTCATTAGC
ACOX1 (M)	CCGTCGAGAAATCGAGAACT	ATTGAGGCCAACAGGTTCCA
CPT1α (M)	ACGTTGGACGAATCGGAACA	GGTGGCCATGACATACTCCC
PPARα (M)	GTGGGTGGTTGAATCGTGAG	GCAGTGGAGTTTGGGTTGG
SCAD (M)	ATGTGCCAGAGGAGCTGAGT	TGATCCACTGTTGCTTCTGC
MCAD (M)	AACTAAACATGGGCCAGCGA	CAGCTGCGACTGTAGGTCTG
LCAD (M)	GCATCAACATCGCAGAGAAA	ACGCTTGCTCTTCCCAAGTA
Apo B (M)	TCACCATTTGCCCTCAACCTA	GAAGGCTCTTTGGAAGTGTAA
MTP (M)	ATCATCATTGGAGCCCTGGT	CATTCTTCAGGGCCAGCA

Gene	Forward primer (5'-3')	Reverse primer (5'-3')
CD36 (M)	TGGTCAAGCCAGCTAGAAA	CCCAGTCTCATTTAGCCAC
FATP1 (M)	CCGTATCCTCACGCATGTGT	CTCCATCGTGTCCTCATTGAC
FATP2 (M)	GATGCCGTGTCCGTCTTTTAC	GACTTCAGACCTCCACGACTC
FATP5 (M)	TCGGATCTGGGAATTCTACG	TTGGTTCTTTCGAACCTTGG
FABP1 (M)	GCCAGGAGAACTTTGAGC	TTGACGACTGCCTTGACT
FFAR1 (M)	CATCACTCTGCCCCTGAAG	AAGGCAAAGACTGGGCAGA
MafK (H)	CTGCGCTCCAAGTACGAGGCG	TCGGTGGACTTGACGATGGTG
MafK (M)	GATGAGCTGGTGTCCATGTCAG	TGTGTCACACGCTTGATGCGAC
β-actin (H, M)	AGCCATGTACGTAGCCATCC	GCTGTGGTGGTGAAGCTGTA

H homo sapiens, *M* mus musculus

Table S4 Mass spectrometry (MS) data of CaM co-immunoprecipitation

Accession	Description	Sum PEP Score	Coverage (%)	# Peptides	# Unique peptides	# Protein groups	# AAs	MW (kD)
Q8BU14	Translocation protein SEC62<br>OS = Mus musculus<br>OX = 10090<br>GN = Sec62<br>PE = 1<br>SV = 1	7.793	7	4	4	1	398	45.6
P58710	L-gulonolactone oxidase<br>OS = Mus musculus<br>OX = 10090<br>GN = Gulo<br>PE = 1<br>SV = 3	7.725	10	5	5	1	440	50.4
Q9EP75	Leukotriene-B4 omega-hydroxylase 3<br>OS = Mus musculus<br>OX = 10090<br>GN = Cyp4f14<br>PE = 1<br>SV = 1	7.669	6	3	3	1	524	59.8
O35465	Peptidyl-prolyl cis-trans isomerase FKBP8<br>OS = Mus musculus<br>OX = 10090<br>GN = Fkbp8<br>PE = 1<br>SV = 2	7.617	8	3	3	1	402	43.5
Q9EST1	Gasdermin-A<br>OS = Mus musculus	7.569	5	3	3	1	446	49.6

Accession	Description	Sum PEP Score	Coverage (%)	# Peptides	# Unique peptides	# Protein groups	# AAs	MW (kD)
	OX = 10090<br>GN = Gsdma<br>PE = 2 SV = 1							
P51855	Glutathione synthetase<br>OS = Mus musculus<br>OX = 10090<br>GN = Gss<br>PE = 1<br>SV = 1	7.568	8	3	3	1	474	52.2
Q91VS7	Microsomal glutathione S-transferase 1<br>OS = Mus musculus<br>OX = 10090<br>GN = Mgst1<br>PE = 1<br>SV = 3	7.555	19	2	2	1	155	17.5
Q80XL6	Acyl-CoA dehydrogenase family member 11<br>OS = Mus musculus<br>OX = 10090<br>GN = Acad11<br>PE = 1<br>SV = 2	7.524	3	2	2	1	779	87.3
Q9QXE7	F-box-like/WD repeat-containing protein TBL1X<br>OS = Mus musculus<br>OX = 10090<br>GN = Tbl1x<br>PE = 1<br>SV = 2	7.491	8	3	3	1	527	56.8
Q9WTU6	Mitogen-activated protein kinase 9	7.48	9	4	4	1	423	48.2

Accession	Description	Sum PEP Score	Coverage (%)	# Peptides	# Unique peptides	# Protein groups	# AAs	MW (kD)
	OS = Mus musculus<br>OX = 10090<br>GN = Mapk9<br>PE = 1<br>SV = 2							
Q8C2Q3	RNA-binding protein 14<br>OS = Mus musculus<br>OX = 10090<br>GN = Rbm14<br>PE = 1<br>SV = 1	7.299	6	3	3	1	669	69.4
Q7TNC4	Putative RNA-binding protein Luc7-like 2<br>OS = Mus musculus<br>OX = 10090<br>GN = Luc7l2<br>PE = 1<br>SV = 1	7.279	8	3	3	1	392	46.6
P61222	ATP-binding cassette sub-family E member 1<br>OS = Mus musculus<br>OX = 10090<br>GN = Abce1<br>PE = 1<br>SV = 1	7.259	7	4	4	1	599	67.3
Q9DBJ3	Brain-specific angiogenesis inhibitor 1-associated protein 2-like protein 1<br>OS = Mus musculus<br>OX = 10090<br>GN = Baiap2l1	7.109	8	4	4	1	514	57.2

Accession	Description	Sum PEP Score	Coverage (%)	# Peptides	# Unique peptides	# Protein groups	# AAs	MW (kD)
	PE = 1<br>SV = 1							
P25444	40S ribosomal protein S2<br>OS = Mus musculus<br>OX = 10090<br>GN = Rps2<br>PE = 1<br>SV = 3	7.065	14	4	4	1	293	31.2
Q8BGS1	Band 4.1-like protein 5<br>OS = Mus musculus<br>OX = 10090<br>GN = Epb41l5<br>PE = 1 SV = 1	7.015	5	3	3	1	731	81.6
Q91Y97	Fructose-bisphosphate aldolase B<br>OS = Mus musculus<br>OX = 10090<br>GN = Aldob<br>PE = 1<br>SV = 3	6.995	10	3	3	1	364	39.5
Q9QYI3	DnaJ homolog subfamily C member 7<br>OS = Mus musculus<br>OX = 10090<br>GN = Dnajc7<br>PE = 1 SV = 2	6.92	5	2	2	1	494	56.4
Q9JLT4	Thioredoxin reductase 2, mitochondrial<br>OS = Mus musculus<br>OX = 10090<br>GN = Txnrd2	6.901	5	2	1	1	524	56.6

Accession	Description	Sum PEP Score	Coverage (%)	# Peptides	# Unique peptides	# Protein groups	# AAs	MW (kD)
	PE = 1<br>SV = 4							
Q9D2Y4	Mixed lineage kinase domain-like protein<br>OS = Mus musculus<br>OX = 10090<br>GN = Mlkl<br>PE = 1<br>SV = 1	6.834	6	2	2	1	472	54.3
P05202	Aspartate aminotransferase, mitochondrial<br>OS = Mus musculus<br>OX = 10090<br>GN = Got2<br>PE = 1<br>SV = 1	6.728	8	3	3	1	430	47.4
P55096	ATP-binding cassette sub-family D member 3<br>OS = Mus musculus<br>OX = 10090<br>GN = Abcd3<br>PE = 1<br>SV = 2	6.712	5	3	3	1	659	75.4
Q8R016	Bleomycin hydrolase<br>OS = Mus musculus<br>OX = 10090<br>GN = Blmh<br>PE = 1<br>SV = 1	6.684	5	2	2	1	455	52.5
P70694	Estradiol 17 beta-dehydrogenase 5<br>OS = Mus musculus	6.67	12	5	5	1	323	37

Accession	Description	Sum PEP Score	Coverage (%)	# Peptides	# Unique peptides	# Protein groups	# AAs	MW (kD)
	OX = 10090							
	GN = Akr1c6							
	PE = 1							
	SV = 1							
Q61048	WW domain-binding protein 4	6.667	7	2	2	1	376	42.1
	OS = Mus musculus							
	OX = 10090							
	GN = Wbp4							
	PE = 1 SV = 4							
P70441	Na^+/H^+ exchange regulatory cofactor NHE-RF1	6.623	12	4	4	1	355	38.6
	OS = Mus musculus							
	OX = 10090							
	GN = Slc9a3r1							
	PE = 1							
	SV = 3							

Table S5 Mass spectrometry (MS) data of DNA pull-down

Accession	Description	Sum PEP score	Coverage (%)	# Peptides	# Unique peptides	# Protein groups	# AAs	MW (kD)
P22361	Hepatocyte nuclear factor 1-alpha<br>OS = Mus musculus<br>OX = 10090<br>GN = Hnf1a<br>PE = 1<br>SV = 2	18.655	10	5	5	1	628	67.2
Q9WU00	Nuclear respiratory factor 1<br>OS = Mus musculus<br>OX = 10090<br>GN = Nrf1<br>PE = 1<br>SV = 2	5.556	4	2	2	1	503	53.5
P42225	Signal transducer and activator of transcription 1<br>OS = Mus musculus<br>OX = 10090<br>GN = Stat1<br>PE = 1<br>SV = 1	4.151	2	1	1	1	749	87.1
Q9R1E0	Forkhead box protein O1<br>OS = Mus musculus<br>OX = 10090<br>GN = FoxO1<br>PE = 1	1.687	2	1	1	1	652	69.5

Accession	Description	Sum PEP score	Coverage (%)	# Peptides	# Unique peptides	# Protein groups	# AAs	MW (kD)
	SV = 2							
Q08639	Transcription factor Dp-1 OS = Mus musculus OX = 10090 GN = Tfdp1 PE = 1 SV = 1	1.001	3	1	1	1	410	45.2
Q64152	Transcription factor Btf3 OS = Mus musculus OX = 10090 GN = Btf3 PE = 1 SV = 3	4.514	9	1	1	1	204	22
Q61827	Transcription factor MafK OS = Mus musculus OX = 10090 GN = Mafk PE = 1 SV=1	1.647	7	1	1	1	156	17.5
